# Supplementary material for: Mapping human pre-rRNA processing and modification at single nucleotide resolution using long read nanopore sequencing
Source: Nat Commun. 2026 Mar 31;17:4658. doi: 10.1038/s41467-026-71164-x (PMC13201660; doi:10.1038/s41467-026-71164-x)
Supplement: Supplementary file 1 — Supplementary Information [file 41467_2026_71164_MOESM1_ESM.pdf]

Supplementary Figures

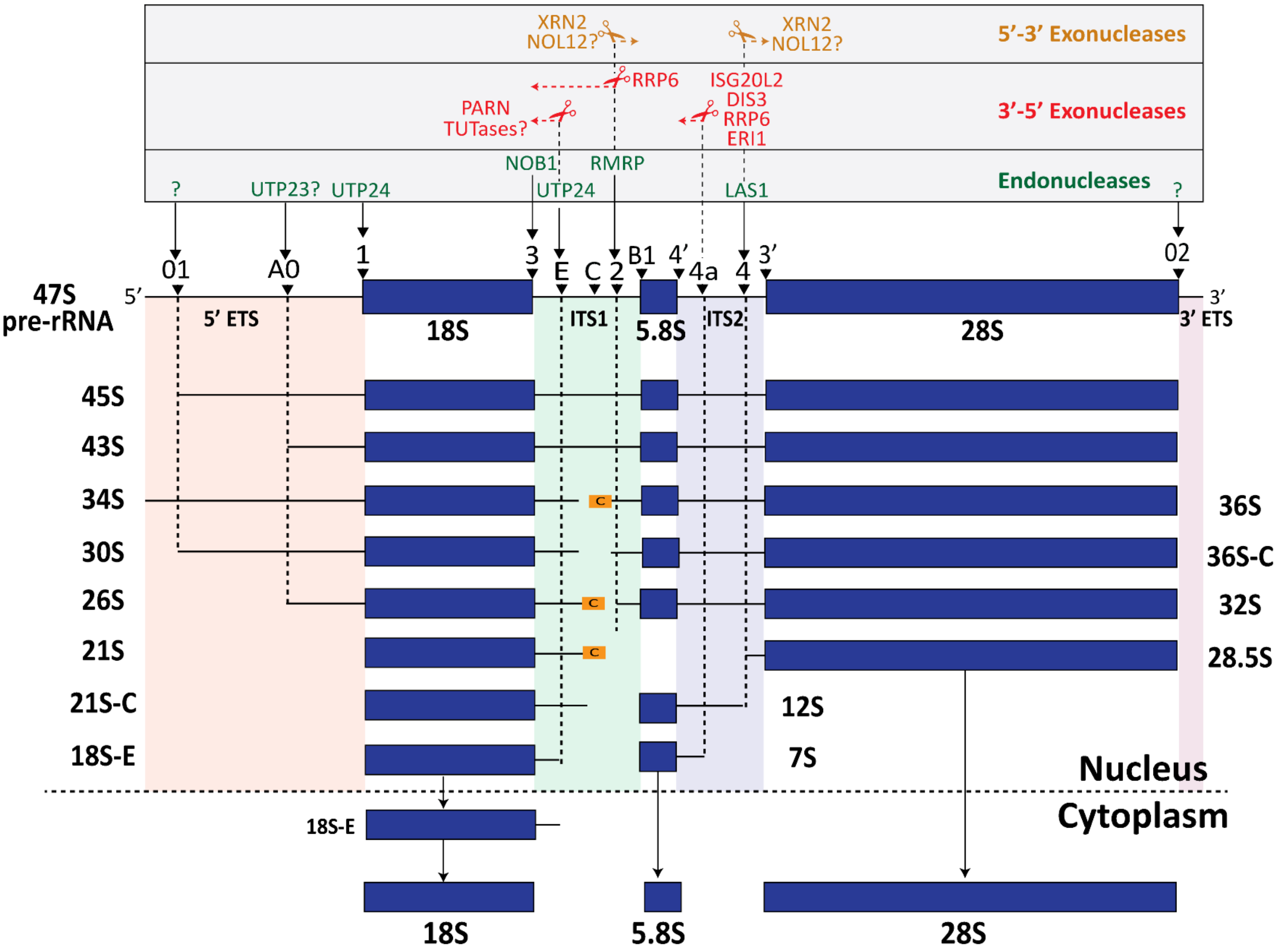

Supplementary Figure S1. Overview of human pre-rRNA processing pathway
Schematic overview of processing sites in the human 47S pre-rRNA, summarizing over two decades of research on rRNA processing within the external transcribed spacers (5′ ETS and 3′ ETS) and internal transcribed spacers (ITS1 and ITS2), shown below the 47S template. Enzymes involved in specific processing steps are indicated: endonucleases (green), 3′-5′ exonucleases (red), 5′-3′ exonucleases (orange), and unknown enzymes (?).
The boundaries for the mature rRNA components are indicated as follows: 18S rRNA (sites 1 and 3), 5.8S rRNA (sites B1 and 4'), and 28S rRNA (sites 3' and 02).
Early precursors include 47S, 45S and 43S. The precursors for 18S rRNA biogenesis include 30S, 26S, 21S, 21S-C, and 18S-E, while the precursors for 28S and 5.8S rRNA include 32S, 28.5S, 12S, and 7S (Ref [1,2,3]). The 36S, 36S-C, and 34S RNAs were associated with dysfunctional subunit biogenesis.

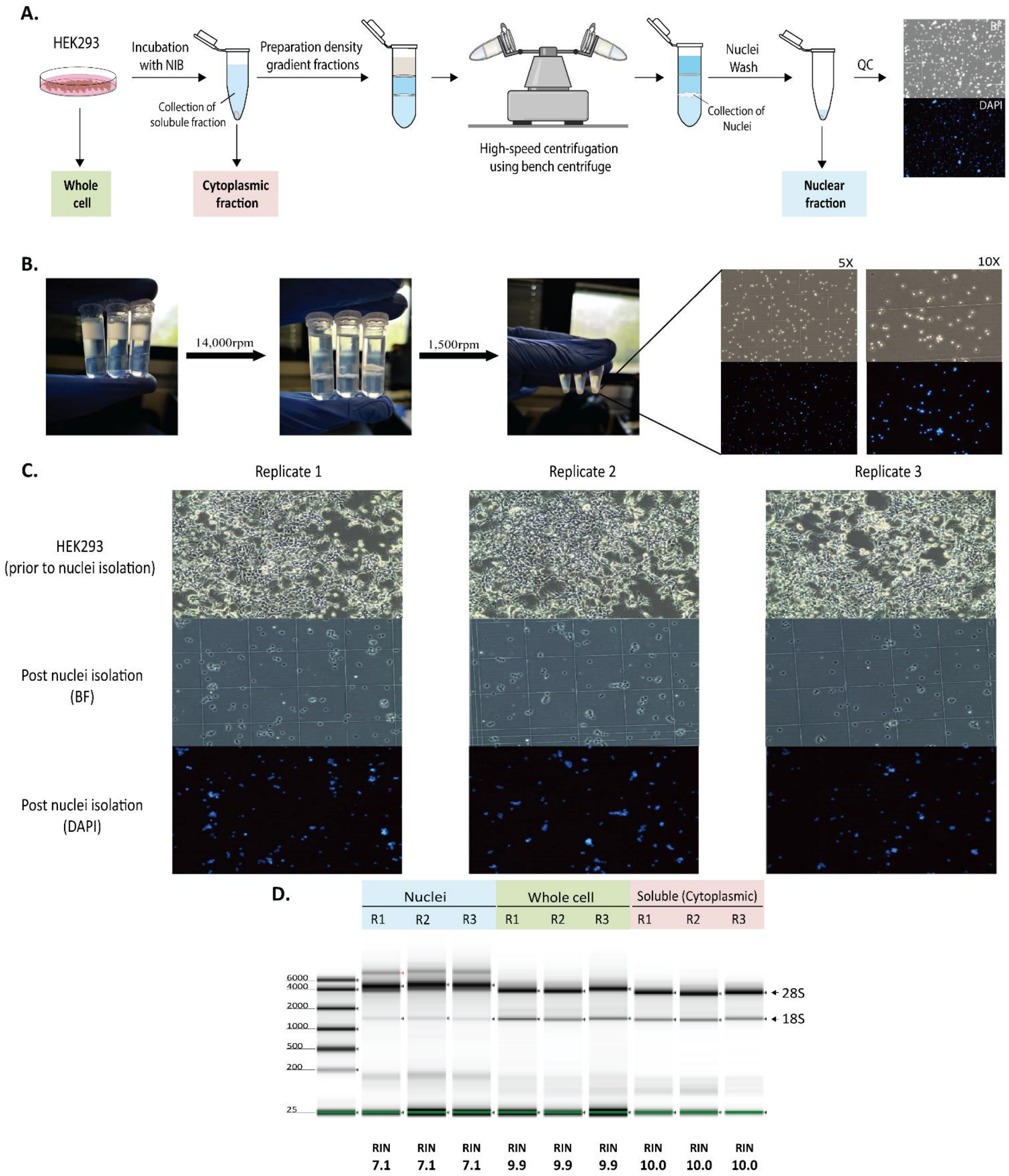

A.
HEK293
Incubation with NIB
Collection of solubule fraction
Preparation density gradient fractions
High-speed centrifugation using bench centrifuge
Nuclei Wash
Collection of Nuclei
QC
BF
DAPI
Whole cell
Cytoplasmic fraction
Nuclear fraction
B.
14,000rpm
1,500rpm
5X
10X
C.
Replicate 1
Replicate 2
Replicate 3
HEK293 (prior to nuclei isolation)
Post nuclei isolation (BF)
Post nuclei isolation (DAPI)
D.
Nuclei
Whole cell
Soluble (Cytoplasmic)
R1 R2 R3 R1 R2 R3 R1 R2 R3
6000
4000
2000
1000
500
200
25
28S
18S
RIN 7.1
RIN 7.1
RIN 7.1
RIN 9.9
RIN 9.9
RIN 9.9
RIN 10.0
RIN 10.0
RIN 10.0

Supplementary Figure S2. Simplified nuclei isolation procedure and quality control steps

A, Detailed schematic diagram of the simplified nuclei isolation procedure. See materials and methods for details. Cells are incubated in nuclei isolation buffer (NIB) for 15 min. Following low speed centrifugation, the soluble fraction (containing the cytoplasmic fraction) is collected and stored for Trizol RNA isolation (light red). Density gradient solutions are prepared from 60% optiprep solution forming three layers where the pellet containing nuclei are placed at the top. Following high-speed centrifugation using a bench centrifuge, the nuclei are collected, washed and assessed under a microscope. Nuclei are then stored in Trizol for RNA isolation (light blue). The whole cell condition is collected at the first step and stored for Trizol RNA isolation as control (light green).

B, Illustration of the 2 ml tubes with density gradient and sample at the top of the tube. Following high-speed centrifugation (~14k rpm), the lower interphase (containing the nuclei) is collected and washed using low-speed centrifugation (~1.5k rpm). A sample from pelleted nuclei is taken, incubated with DAPI, and analyzed under bright field microscopy with UV filter to visualize the quality of the nuclei.

C, Representative microscope image of HEK293 cells (top) prior nuclei isolation, bright field post nuclei isolation (middle) and DAPI stained nuclei post nuclei isolation (bottom).

D, Tape-station analysis of RNA profiles of cytoplasmic (light red), whole cell (light green) and nuclear (blue) samples (n=3 samples each). RIN, RNA integrity number.

Supplementary Figure S3. Northern blot analyses performed throughout the study using the indicated probes across total (T), cytoplasmic (C), and nuclear (N) fractions. The respective probes are shown at the top.

5' ETS probing (LD1844)

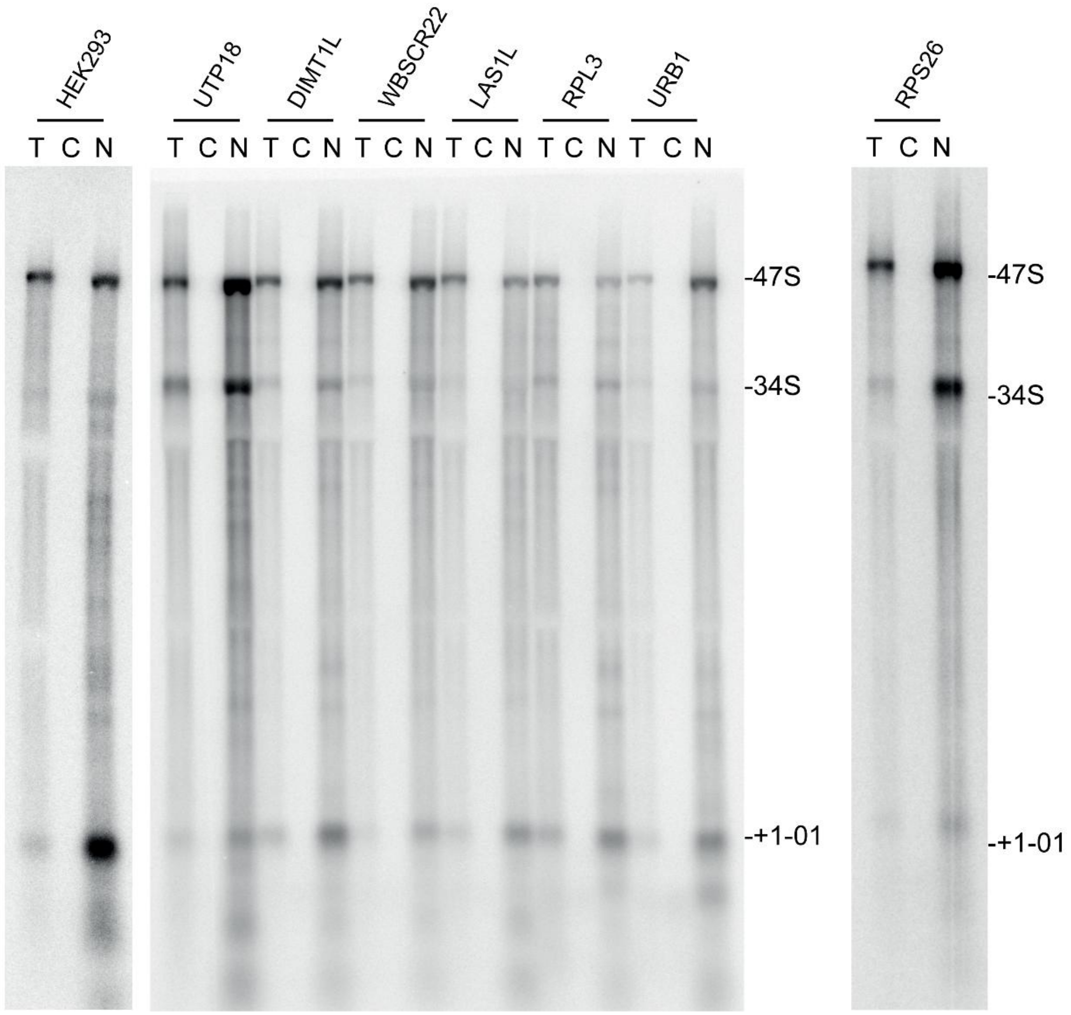

(Figure continues next page)

ITS1 probing (LD2122)

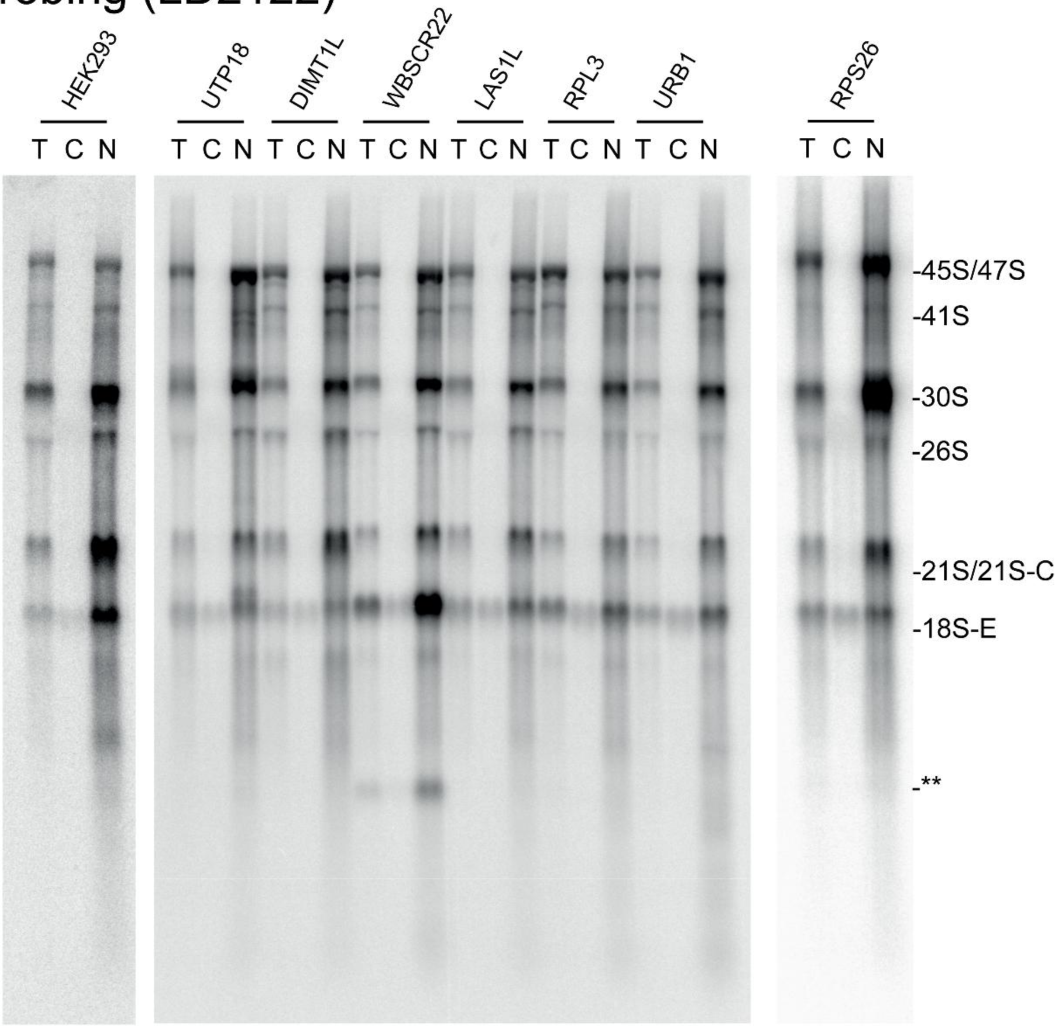

(**) see Zorbas *et al.* (2015) doi: 10.1091/mbc.E15-02-0073

(Figure continues next page)

ITS2 probing (LD1828)

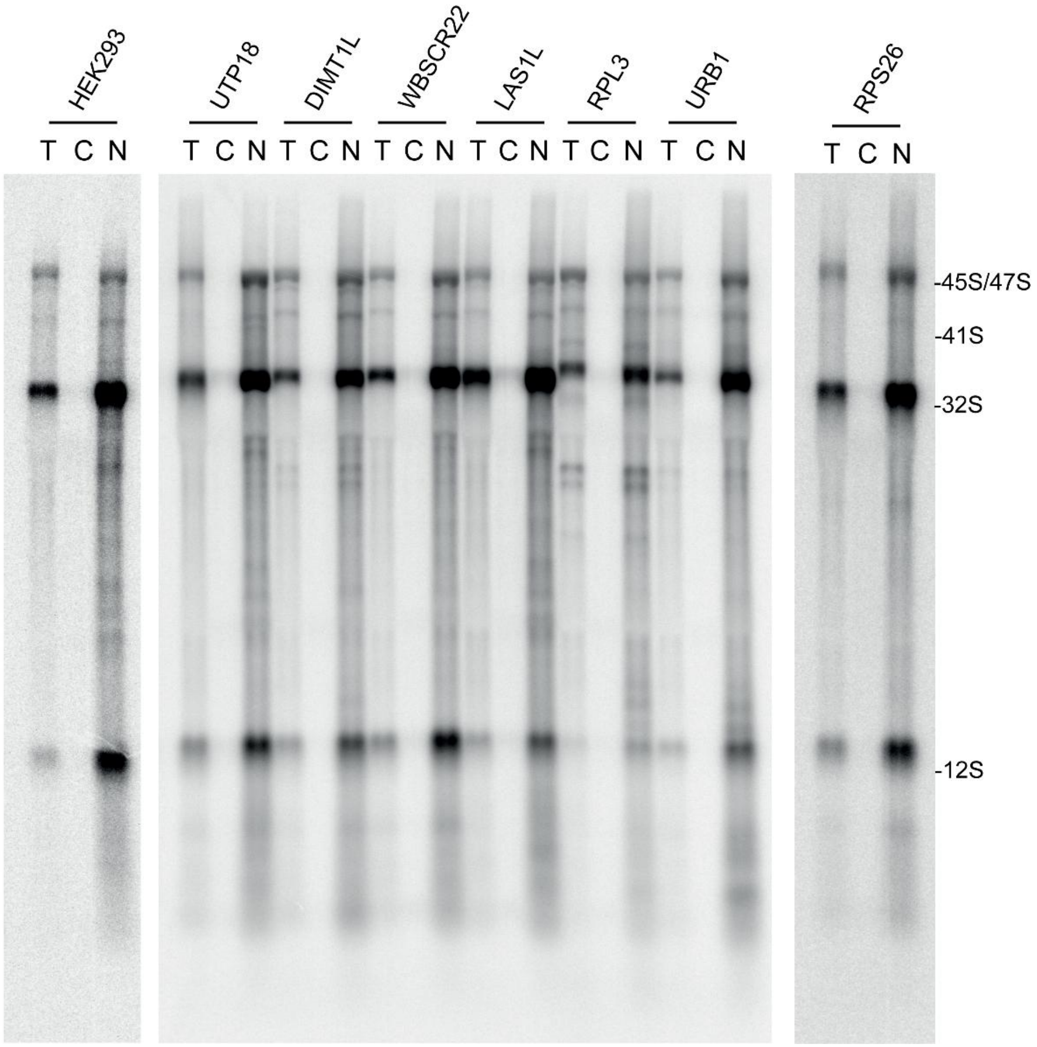

(Figure continues next page)

3’ ETS probing (LD2612)

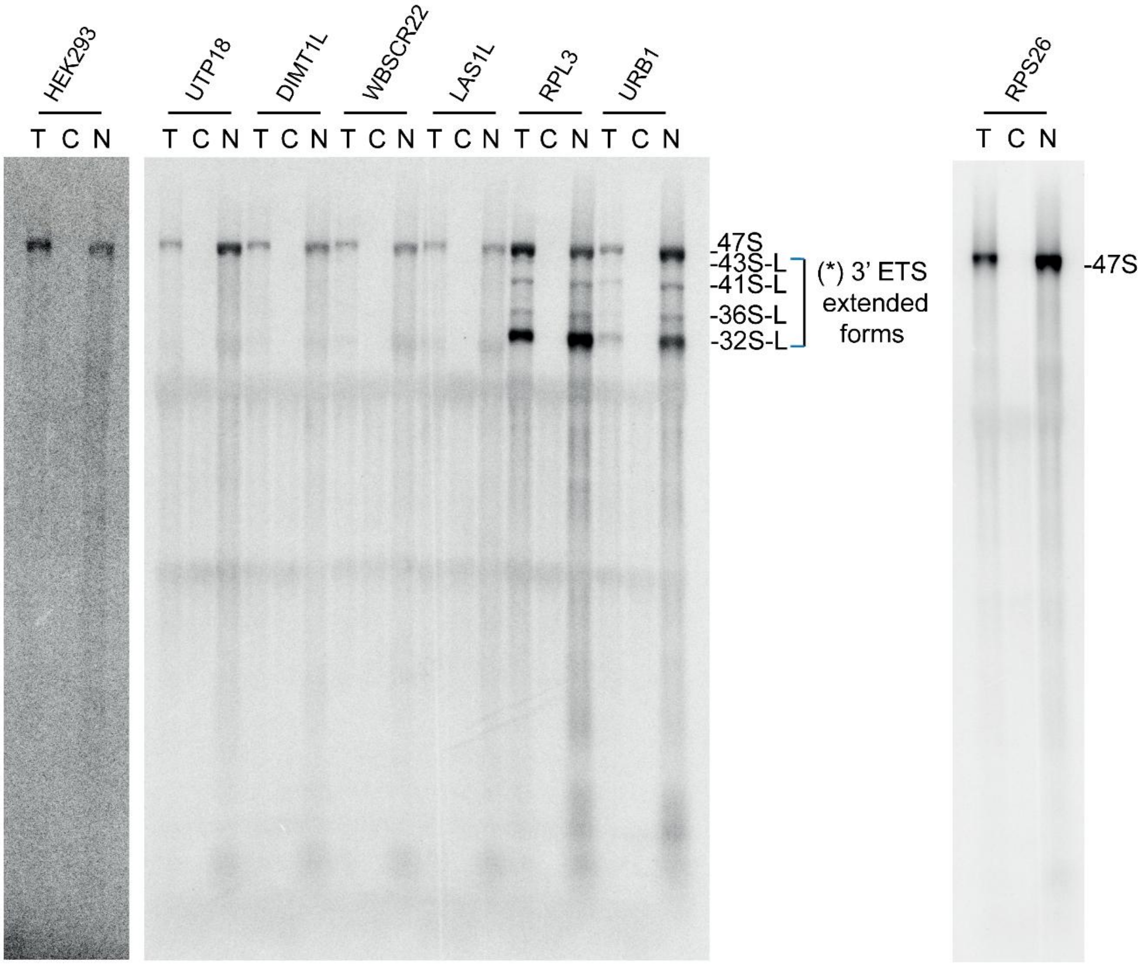

(*) see Langhendries *et al.* (2016) doi: 10.18632/oncotarget.11148

(Figure continues next page)

Ethidium bromide staining

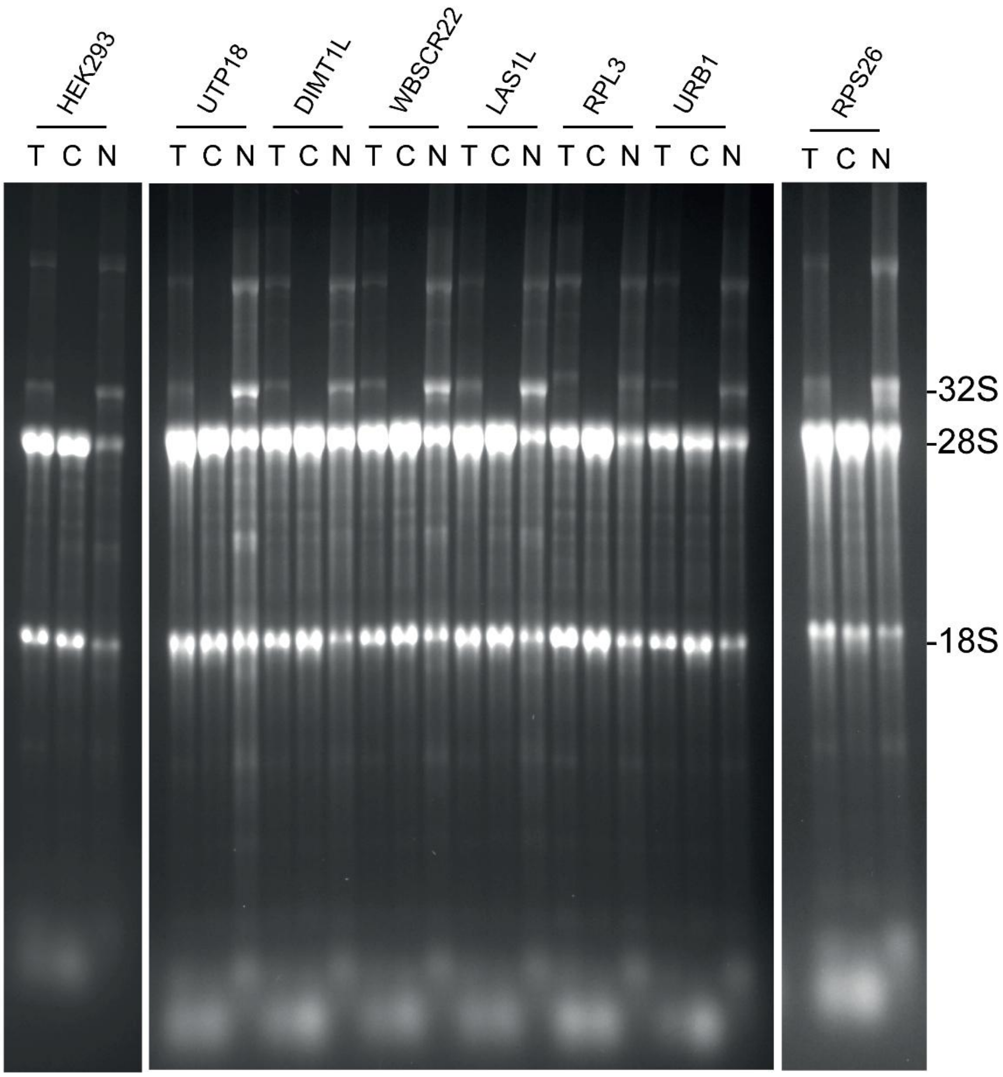

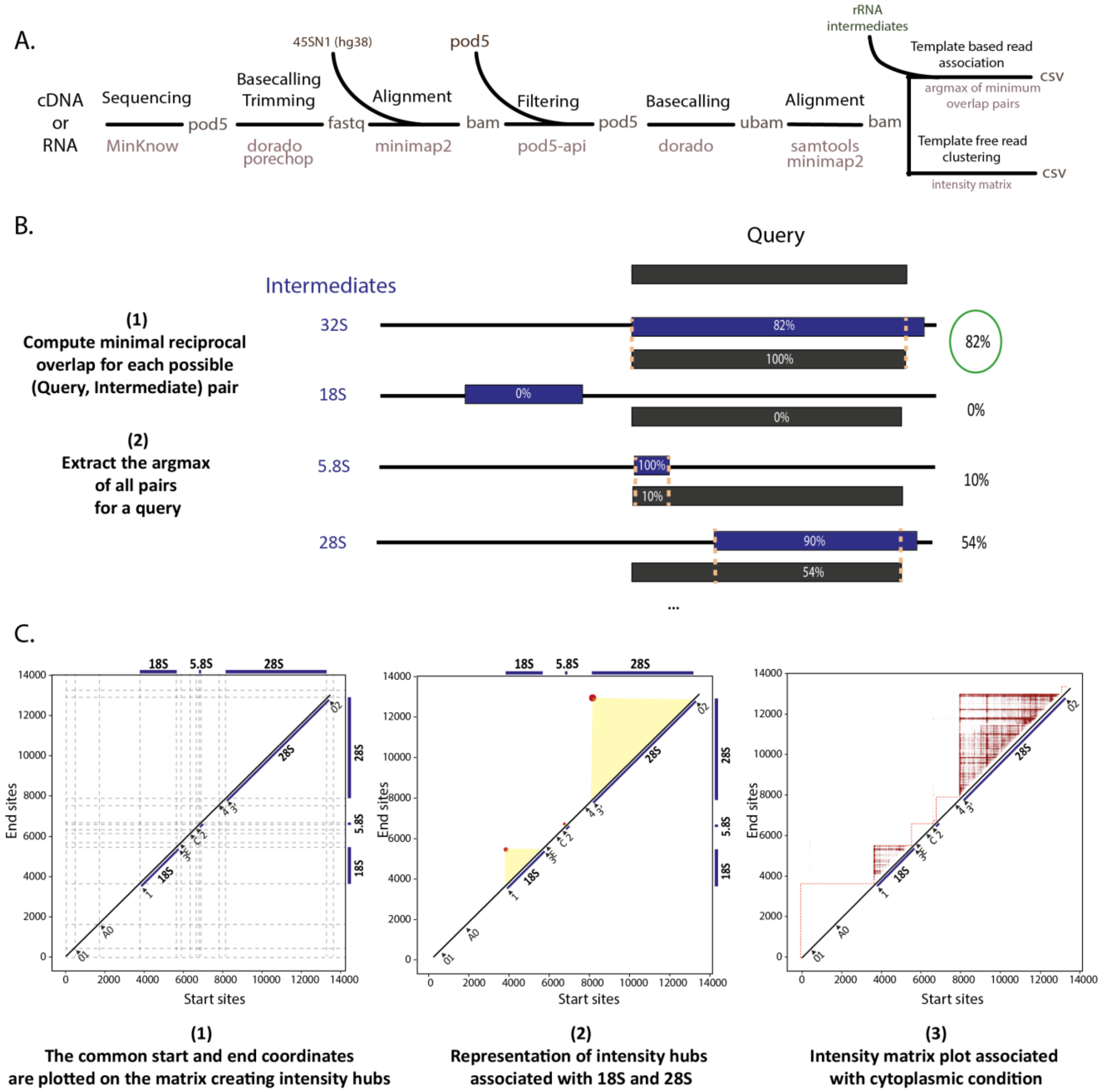

Supplementary Figure S4. Overview of the NanoRibolyzer bioinformatic pipeline

A, NanoRibolyzer pipeline. Reads of input pod5 become basecalled and trimmed. Alignment to 45SN1 of hg38 is used to extract reads aligning to ribosomal RNA. Extracted reads become rebasecalled to perform polyA-estimation and modification detection. Rebasecalled reads are realigned to 45SN1 of hg38. Template-free and template-based read association analysis is performed on realigned reads.

B, Supervised or template-based approach. Minimal reciprocal overlap (MRO) between query read and all literature-based intermediates is computed based on alignment start and end sites of the query. The query read is associated with the intermediate with the maximal query/intermediate MRO. Formal: I={Intermediates}, Q={Query reads}, A={Associated query reads}, $Q_n \in Q$, A_n=argmax(min(overlap(I_i,Q_n),overlap(Q_n,I_i)) $\forall I_i \in I$).

C, unsupervised or template-free approach. A 2-D matrix at the length of the RNA45SN1 template is constructed. Augmentation of reads by start and end site coordinate according to the alignment reveals intensity hubs. Intensity hubs are min-max normalized with a contrast enhancement of 2%.

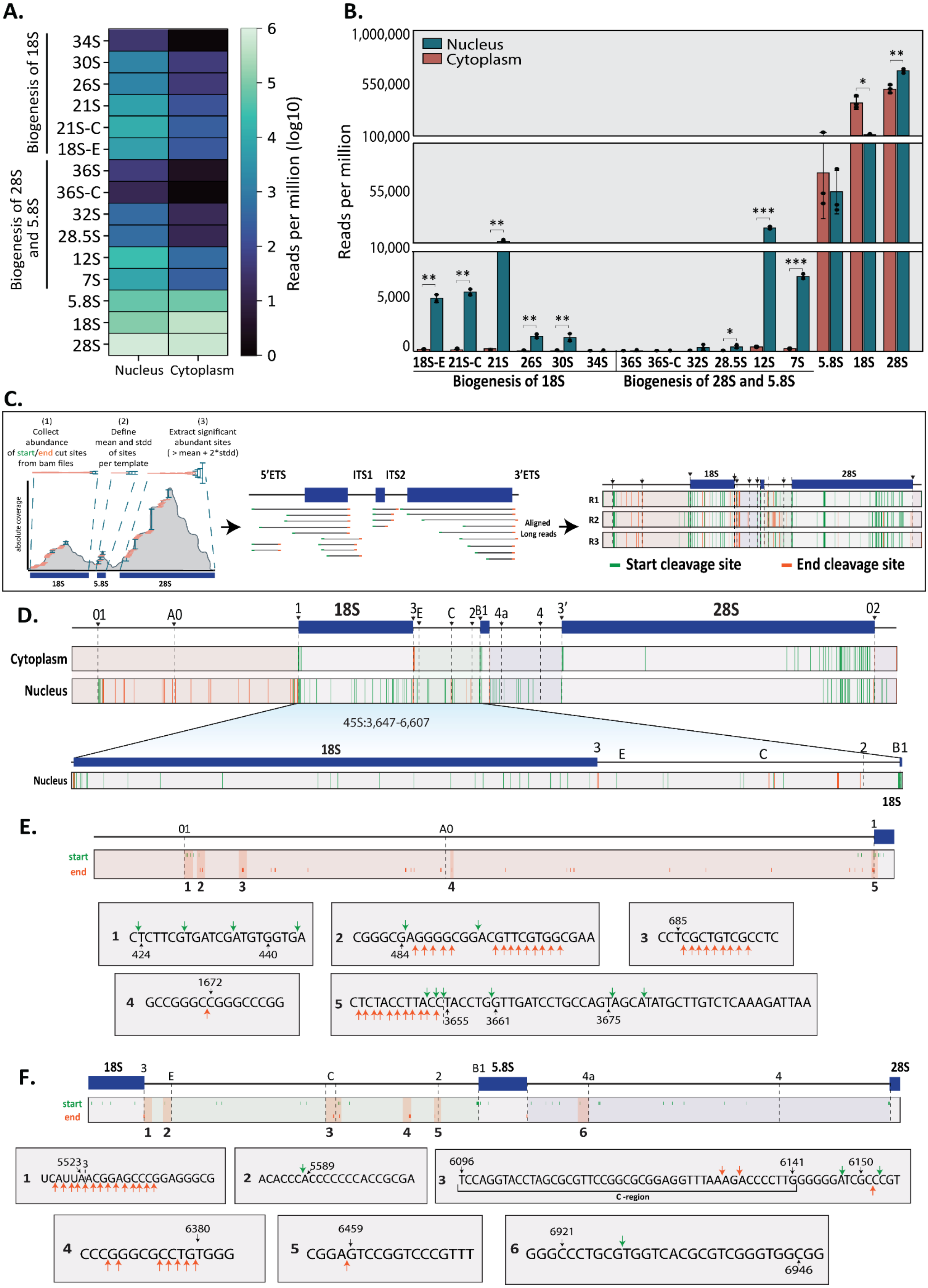

A.
Biogenesis of 18S
Biogenesis of 28S and 5.8S
34S
30S
26S
21S
21S-C
18S-E
36S
36S-C
32S
28.5S
12S
7S
5.8S
18S
28S
Nucleus
Cytoplasm
Reads per million (log10)
B.
Nucleus
Cytoplasm
Reads per million
1,000,000
550,000
100,000
55,000
10,000
5,000
18S-E 21S-C 21S 26S 30S 34S 36S 36S-C 32S 28.5S 12S 7S 5.8S 18S 28S
Biogenesis of 18S
Biogenesis of 28S and 5.8S
C.
(1) Collect abundance of start/end cut sites from bam files
(2) Define mean and stdd of sites per template
(3) Extract significant abundant sites (> mean + 2*stdd)
absolute coverage
18S
5.8S
28S
5'ETS
ITS1
ITS2
3'ETS
Aligned Long reads
R1
R2
R3
Start cleavage site
End cleavage site
D.
01
A0
1
18S
3
E
C
2
B1
4a
4
3'
28S
02
Cytoplasm
Nucleus
45S:3,647-6,607
18S
Nucleus
E.
start
end
01
A0
1
1 2 3 4 5
1 CTCTTCGTGATCGATGTGGTGA 424 440
2 CGGGCGAGGGGCGGACGTTCGTGGCGAA 484
3 CCTCGCTGTCGCCTC 685
4 GCCGGGCCGGGCCCGG 1672
5 CTCTACCTTACCTACCTGGTTGATCCTGCCAGTAGCATATGCTTGTCTCAAAGATTAA 3655 3661 3675
F.
18S
5.8S
28S
start
end
1 2 3 4 5 6
1 UCAUUAACGGAGCCCGGAGGGCG 5523
2 ACACCCACCCCCCCACCGCGA 5589
3 TCCAGGTACCTAGCGCGTTCCGGCGCGGAGGTTTAAAGACCCCTTGGGGGGATCGCCCGT 6096 6141 6150
C-region
4 CCCGGGCGCCTGTGGG 6380
5 CGGAGTCCGGTCCCGTTT 6459
6 GGGCCCTGCGTGGTCACGCGTCGGGTGGCGG 6921 6946

Supplementary Figure S5. NanoRibolyzer's supervised approach for the quantification of pre-rRNA intermediates and the identification of cleavage sites

A, Heatmap illustrating pre-/rRNA abundance in nuclear and cytoplasmic fractions, represented as log10 reads per million (averaged across three replicates).

B, Quantitative comparison of pre-rRNA intermediates and mature rRNA between nuclear and cytoplasmic fractions (n=3 samples each). Histograms display mean reads per million ± SD. Statistical significance was determined using a t-test between nucleus and cytoplasm conditions, with $*p < 0.05$, $**p < 0.01$, $***p < 0.001$.

C, Extraction of prevalent cleavage sites strategy. Prevalent cleavage sites are extracted from the template-based precursors by collecting their start (green) and end (orange) sites for each precursor. The mean and standard deviation of these sites are calculated, and significant cleavage sites are those with abundances at least two standard deviations above the mean. The results are then saved in TSV and BED formats for visualization.

D, Visual representation of prevalent start (green) and end (orange) cleavage sites across the 47S rRNA in cytoplasm (top) and nucleus (bottom). Close-up view of prevalent cleavage sites at the 18S and ITS1 regions are shown below. Putative processing sites and mature rRNA sequences are annotated at the top of each respective figure. The coordinates spanning the region on the 47S rRNA are displayed above.

E-F, Detailed annotation of cleavage sites at single nucleotide resolution. Selected sites near bona fide processing sites within the 5' ETS (E), ITS1 and ITS2 (F), are highlighted, numbered, and the corresponding numbers are mapped to the actual sequence. The annotated sequences include nucleotide positions (black arrows), as well as start (green arrows) and end (orange arrows) cleavage sites, derived from cDNA data. Each cleavage site displayed was detected in at least two out of three replicates.

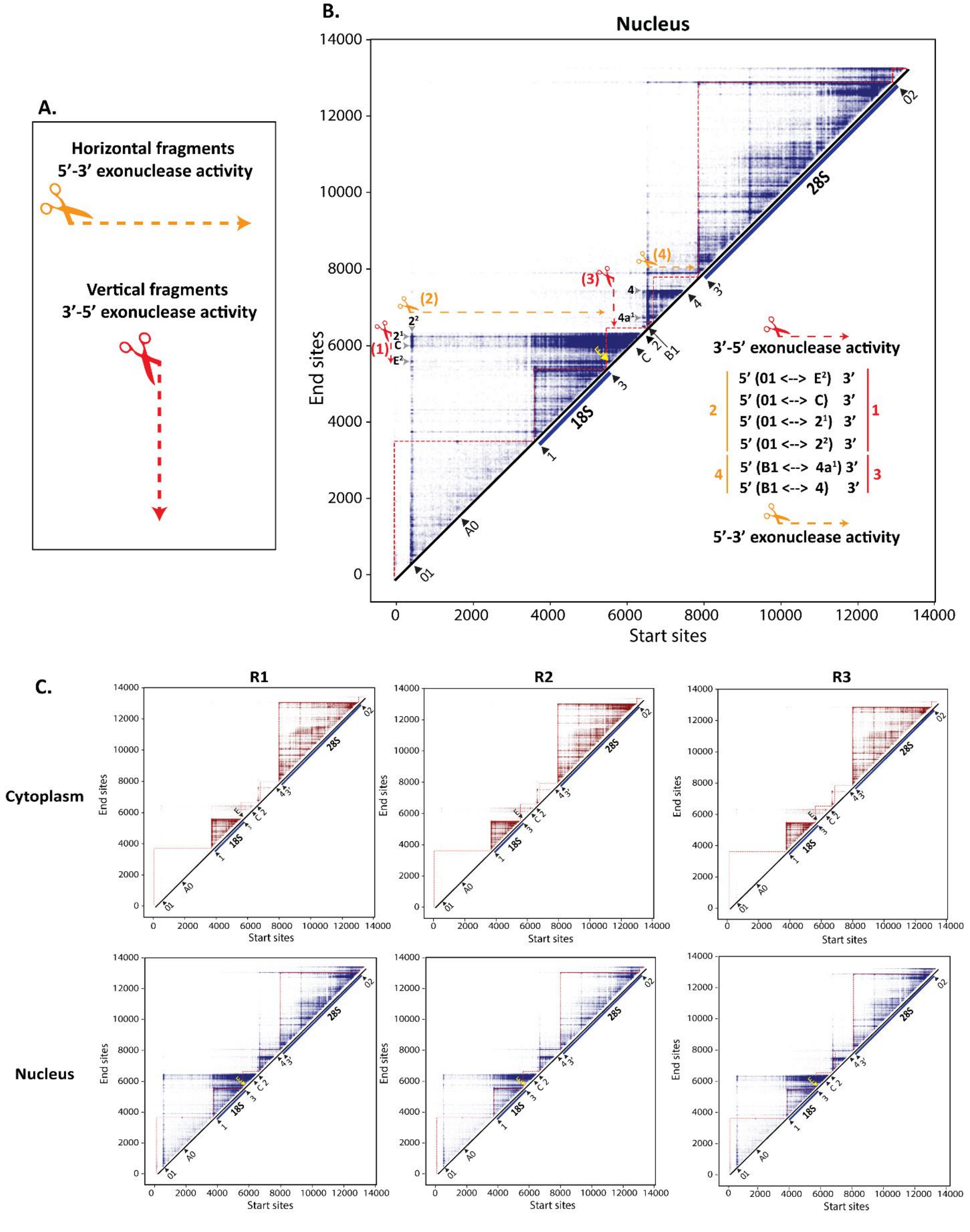

Supplementary Figure S6. NanoRibolyzer's unsupervised approach for the detection of novel intermediates and characterization of exonucleolytic cleavage events using the intensity matrix

A, The intensity matrix provides a clear visualization of exonucleolytic cleavage patterns: Horizontal fragmentation indicates a retained end processing site, with altered start sites, consistent with 5′-3′ exonuclease activity (orange). Vertical fragmentation indicates a retained start processing site, with altered end sites, consistent with 3′-5′ exonuclease activity (red).

B, Retained end processing sites are marked in black within the intensity matrix in the nuclear fraction. The 5' and 3' processing sites are indicated on the right with the directionality of exonuclease acitvity colored appropriately.

C, Intensity matrices of cytoplasm (top) and nuclei (bottom) conditions (n=3 samples each).

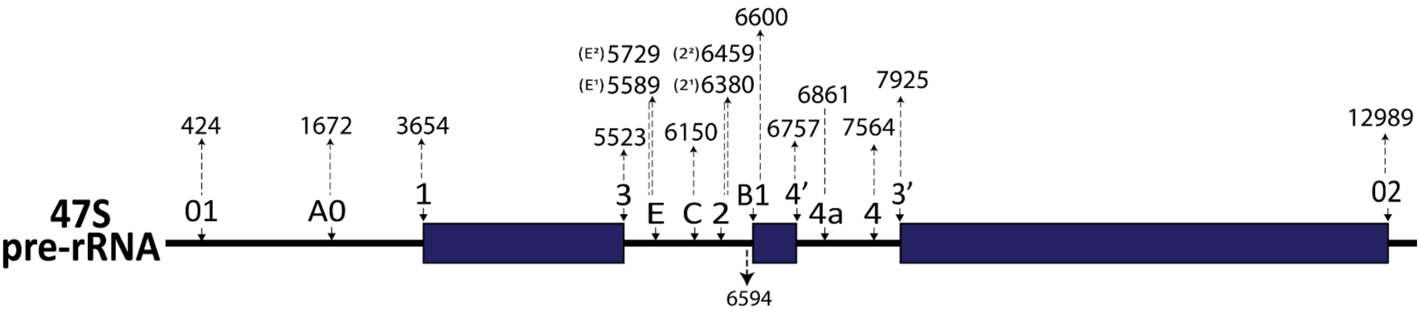

Supplementary Figure S7. Detailed description of human ribosomal processing sites

(1) For **site 01**, the start site of the associated intensity hub maps to position C^{424}^T (cleavage site marked by ^), and two additional consistent start sites nearby, G^{429}^T and G^{443}^A (Fig S5E, Source Data 2),which confirms previous findings[4] (Fig 3A; Hubs n° 1b, 2, 3, and 4).

(2) For **site A0**, we identified a hub with a start site at ^{1672}C^G, located 20 nucleotides downstream of the proposed processing site[5] (Fig 3A; Hub n° 1a).

(3) For **site 1**, marking the 5' end of 18S, we detected multiple hubs containing the start site ^{3654}C^T (Fig 3A; Hubs n° 5a, 5b, 5c, and 5d)

(4) For **site 3**, corresponding to the 3' end of 18S, we confirmed site ^{5523}A^A (Fig 3A**;** Hub n° 5d).

(5) For **site E**, interestingly, we identified two distinct end points: one we refer to as "**E^1**", located 11 nucleotides downstream of the previously reported site E (+78 nt extensions beyond the mature 18S end, Refs [6,7]), with multiple cleavages observed at coordinate C^A^{5589}, and the other we refer to as "**E^2**", located in an AG-rich region, with reproducible end cleavages around G^A^{5729} (Fig 3A and S6A-B ; Hubs n° 1b and 5c).

(6) For **site C**, we identified an end point located at site C^G^{6150}, 8 nucleotides downstream of the previously described conserved "region C" (Ref.[6]) (Fig 3A; Hub n° 2).

(7) For **site 2**, our analysis also revealed two distinct intensity hubs: site "2^1", located at G^T^{6380}, and site "2^2", positioned near A^G^{6459}, in agreement with the former observations [8,9] (Fig 3A and S6A-B; Hubs n° 3, 4, 5a, and 5b). These sites could also be observed in the coverage profiles upstream the putative site 2 (Fig 1C-D). These findings reveal two potential precursors associated with 21S, as previously suggested[10].

(8) For site **B1**, we detected the intensity hub precisely at the expected 5' end of 5.8S, at ^{5600}A^CGA, defining the short form of 5.8S, the $5.8S_S$ (Fig 3A; Hub n° 6a, 8, 9, and 10). Interestingly, we identified an intensity hub six nucleotides upstream of the 5.8S start site at position ^{6594}C^C (Fig 3A; annotated as ~B1 in hubs 6b and 7). This site most likely corresponds to the previously described 5' extended version of 5.8S (both coexist in cells, see e.g. [11]), defining the long form of 5.8S, the $5.8S_L$ ([8,11,12]).

(9) For **site 4'**, corresponding to the end site of 5.8S, we confirmed the end point located at site T^T^{6757}(Fig 3A; Hub n° 6a**)**.

(10) For the still poorly characterized **site 4a** in ITS2 (Refs [1,2,3]), we reveal a very clear end point intensity hub located at AGA^C^{6861} (Fig 3A; Hub n° 6b).

(11) For the uncharacterized **site 4**, we identified a distinct intensity hub at the end coordinate T^{7564}GT, defining its cleavage site (Fig 3A; Hubs n° 7-8).

(12) Next, at **site 3'**, marking the start of 28S, we confirmed the start point located at site ^{7925}A^C **(**Fig 3A; Hub n° 11).

(13) And lastly, for **site 02**, marking the end of 28S, we confirmed the end point located at site ^{12990}C^C (Fig 3A; Hub n° 11).

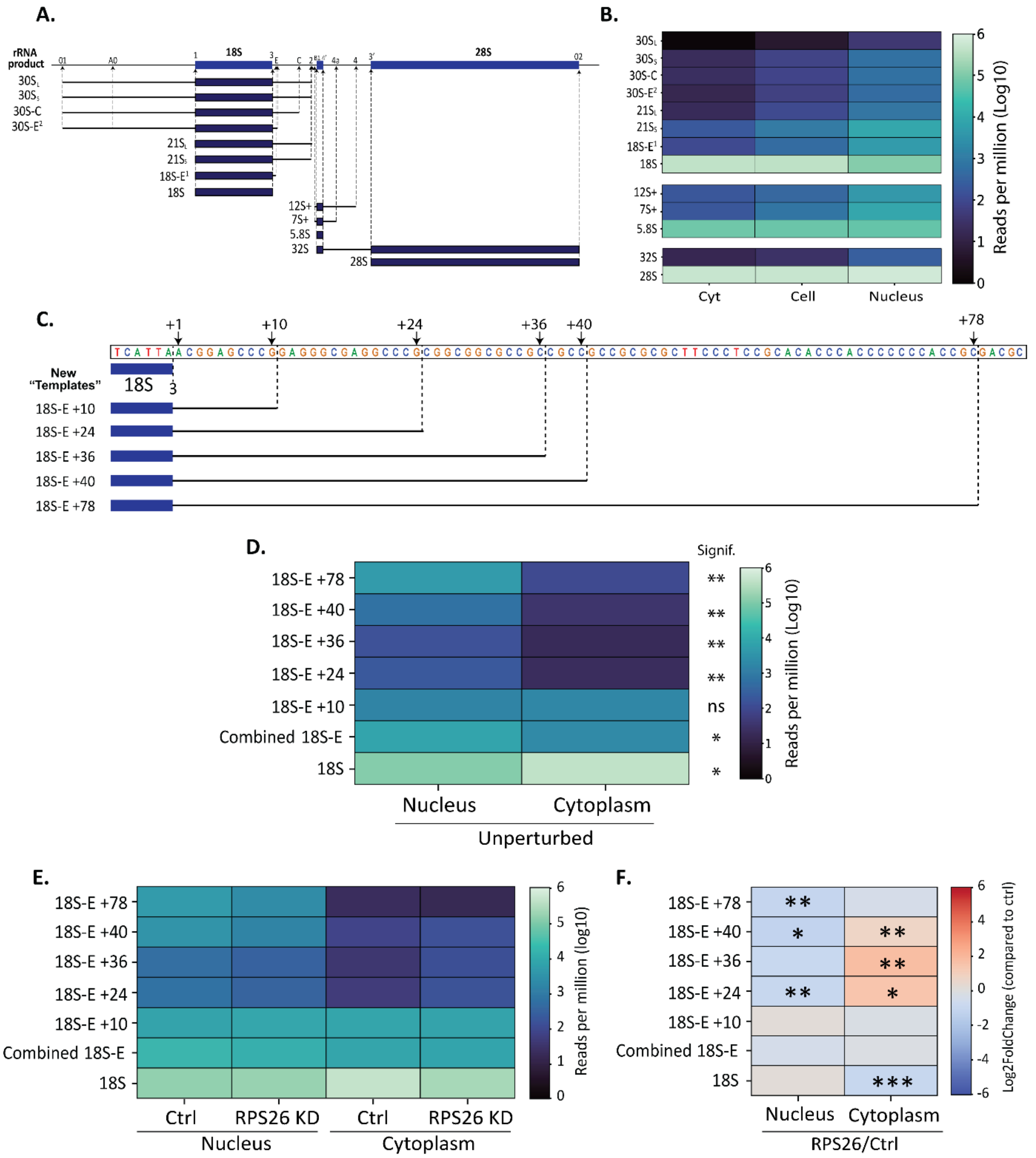

Supplementary Figure S8. Quantification of newly detected pre-rRNA intermediates and distinct 18S-E subtypes

A, Illustration of the identified rRNA products in nucleus, as derived from the intensity hubs table shown in 3A.

B, Quantification of the newly detected pre-rRNA products in Nuclear, whole cell and cytoplasmic fractions using the template-fitted approach.

C, Illustration of the exonucleolytic trimming sites as previously reported[6] including: +10, +24, +36, +40, and +78 nt extensions beyond the mature 18S (termed 18S-E sub-variants). These were used as new templates for the template-fitted approach.

D, Quantification of the 18S-E sub-variants in the nucleus and cytoplasm (n=3 samples each). Statistical significance was determined using a two-tailed paired t-test between nuclear and cytoplasmic conditions, with *p < 0.05, **p < 0.01, ns= non-significant.

E, Quantification of the 18S-E sub-types in the nucleus and cytoplasm of ctrl and RPS26 depelted samples (n=3 samples each).

F, Log2FoldChange of quantified pre-rRNA intermediates and mature rRNAs in the nucleus and cytoplasm of RPS26 knockdown compared to control (n=3 samples each).

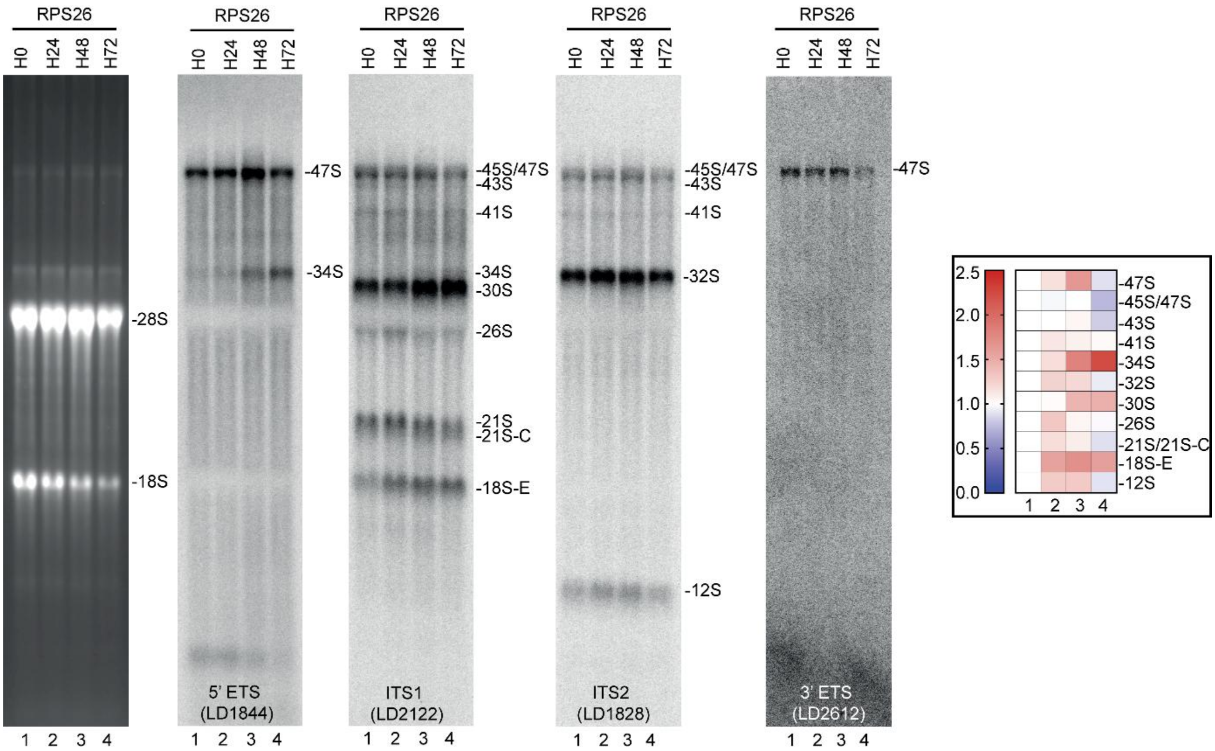

Supplementary Figure S9. Northern blots showing the optimal depletion time points for RPS26
Assessment of intermediate accumulation following processing perturbation using northern blotting. RPS26 was depleted in HEK293 cells in a time course (from 24 to up to 72 h) to identify the best time point for NanoRibolyzer analysis.

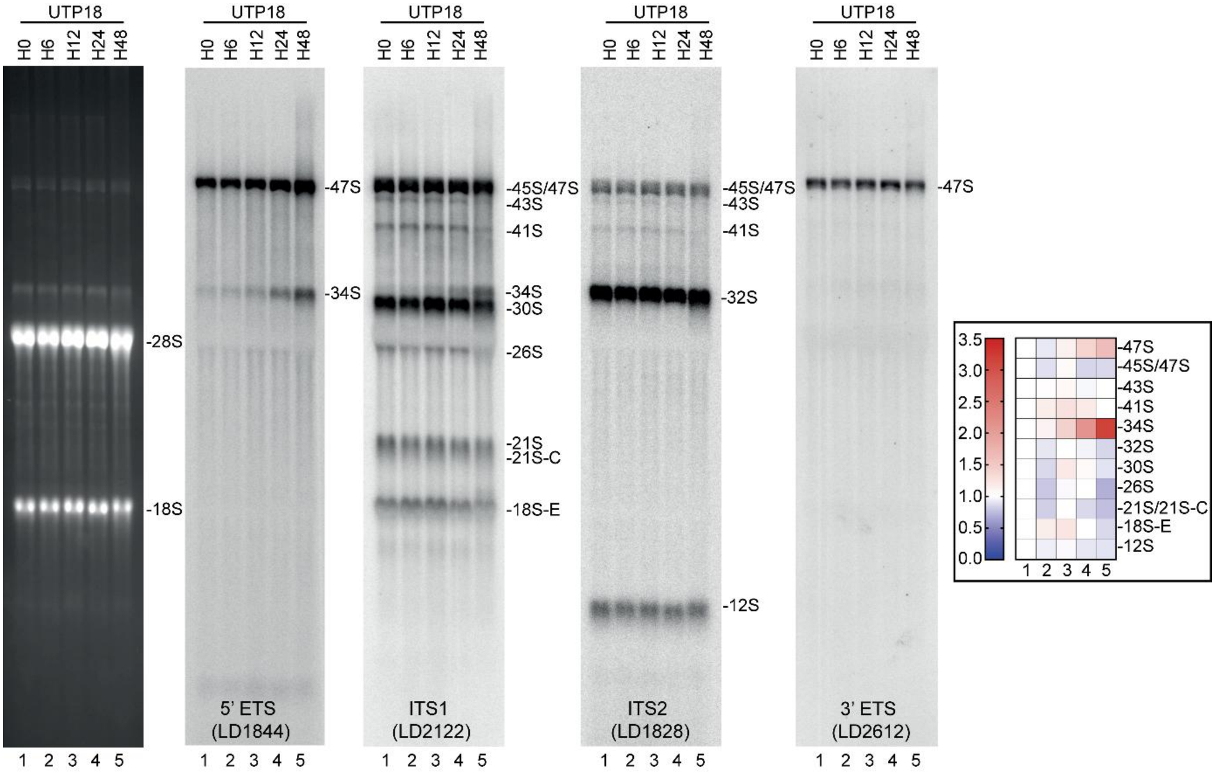

Supplementary Figure S10. Northern blots showing the optimal depletion time points for UTP18
Assessment of intermediate accumulation following processing perturbation using northern blotting. UTP18 was depleted in HEK293 cells in a time course (from 24 to up to 72 h) to identify the best time point for NanoRibolyzer analysis.

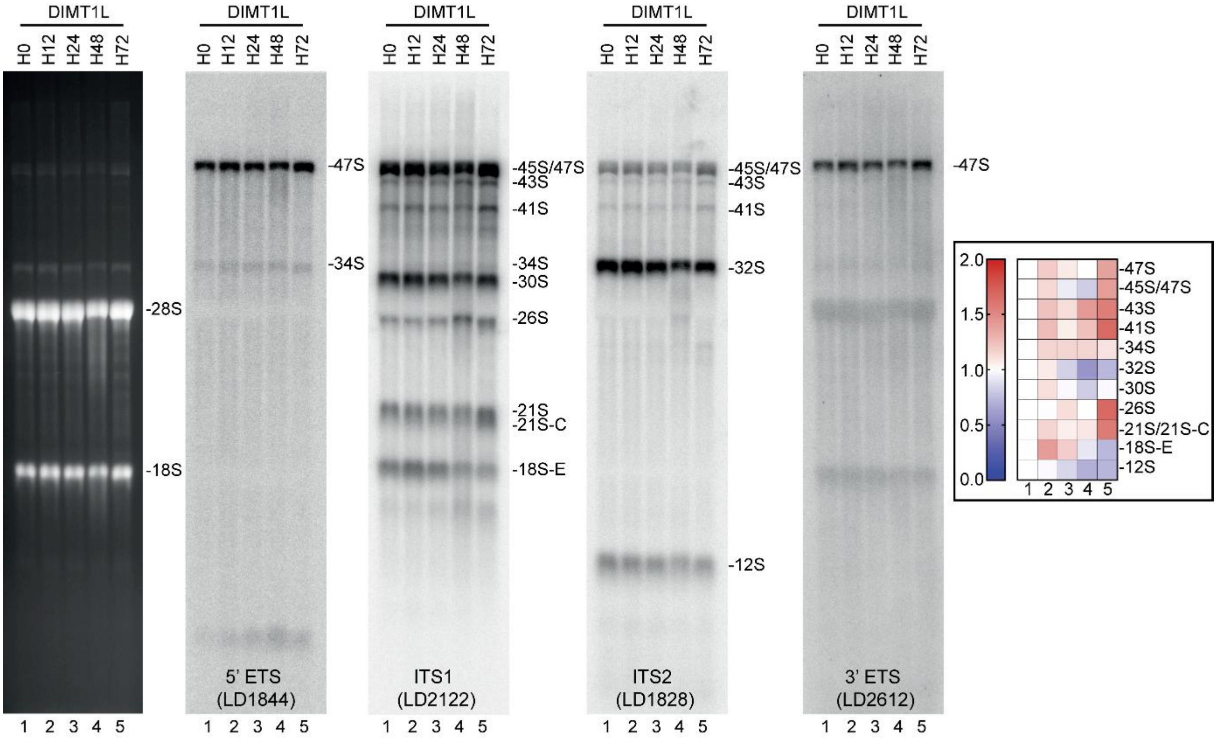

Supplementary Figure S11. Northern blots showing the optimal depletion time points for DIMT1L

Assessment of intermediate accumulation following processing perturbation using northern blotting. DIMT1L was depleted in HEK293 cells in a time course (from 24 to up to 72 h) to identify the best time point for NanoRibolyzer analysis.

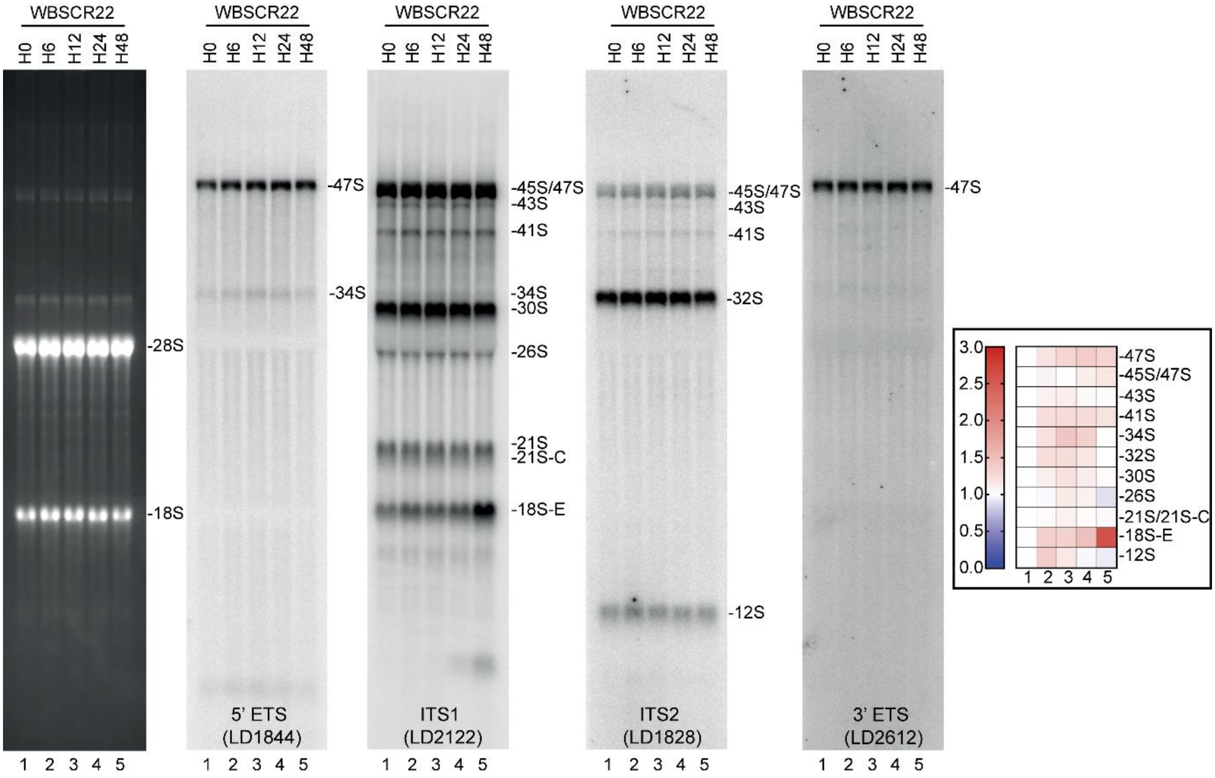

Supplementary Figure S12. Northern blots showing the optimal depletion time points for WBSCR22

Assessment of intermediate accumulation following processing perturbation using northern blotting. WBSCR22 was depleted in HEK293 cells in a time course (from 24 to up to 72 h) to identify the best time point for NanoRibolyzer analysis.

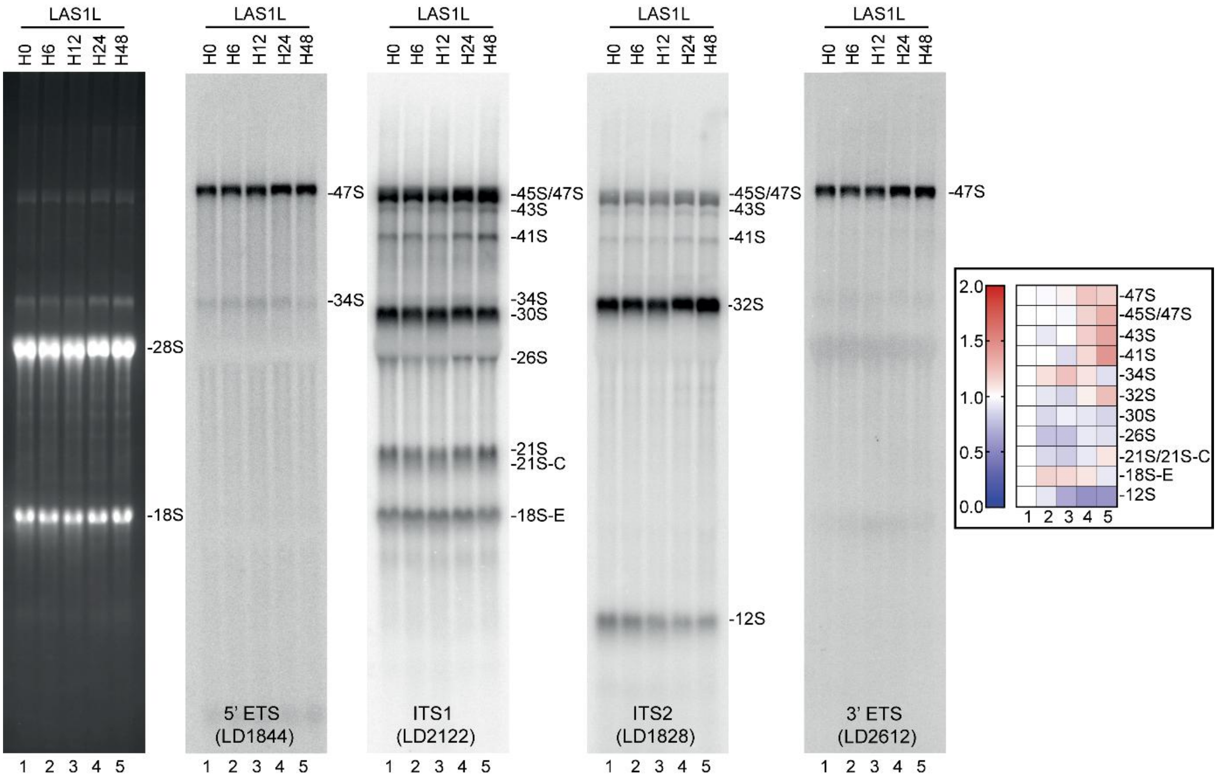

Supplementary Figure S13. Northern blots showing the optimal depletion time points for LAS1L

Assessment of intermediate accumulation following processing perturbation using northern blotting. LAS1L was depleted in HEK293 cells in a time course (from 24 to up to 72 h) to identify the best time point for NanoRibolyzer analysis.

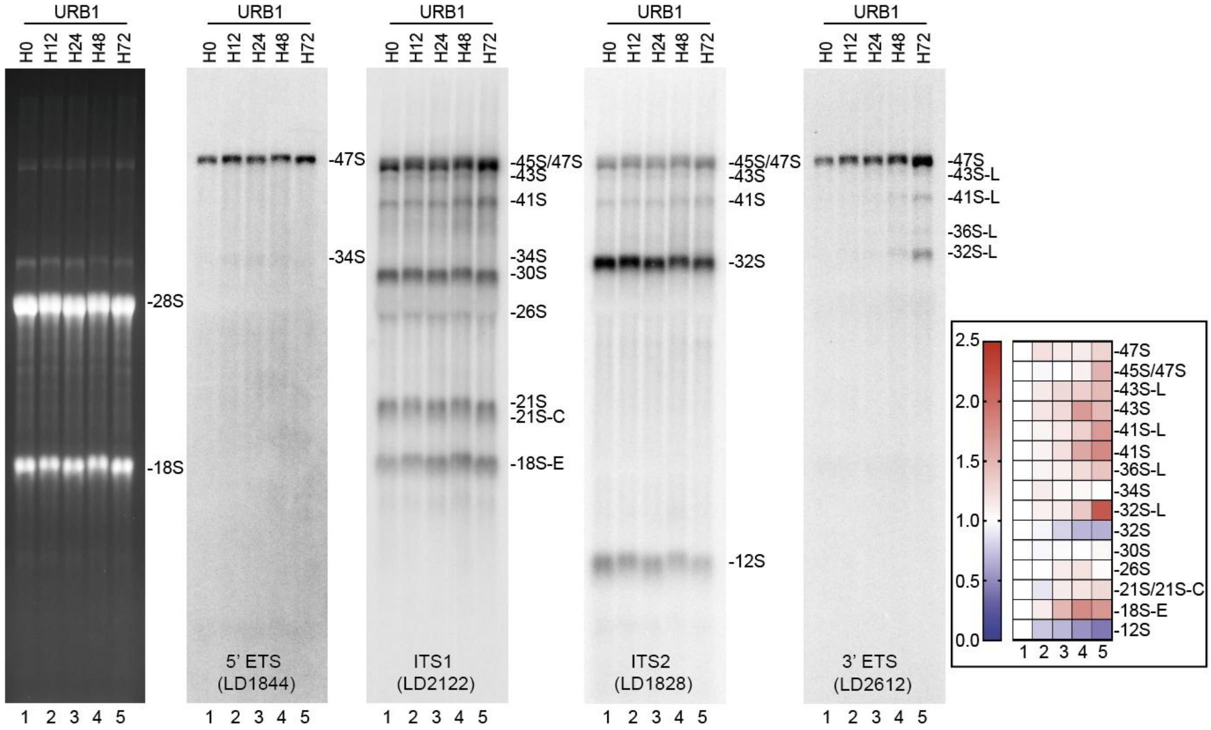

Supplementary Figure S14. Northern blots showing the optimal depletion time points for URB1
Assessment of intermediate accumulation following processing perturbation using northern blotting. URB1 was depleted in HEK293 cells in a time course (from 24 to up to 72 h) to identify the best time point for NanoRibolyzer analysis.

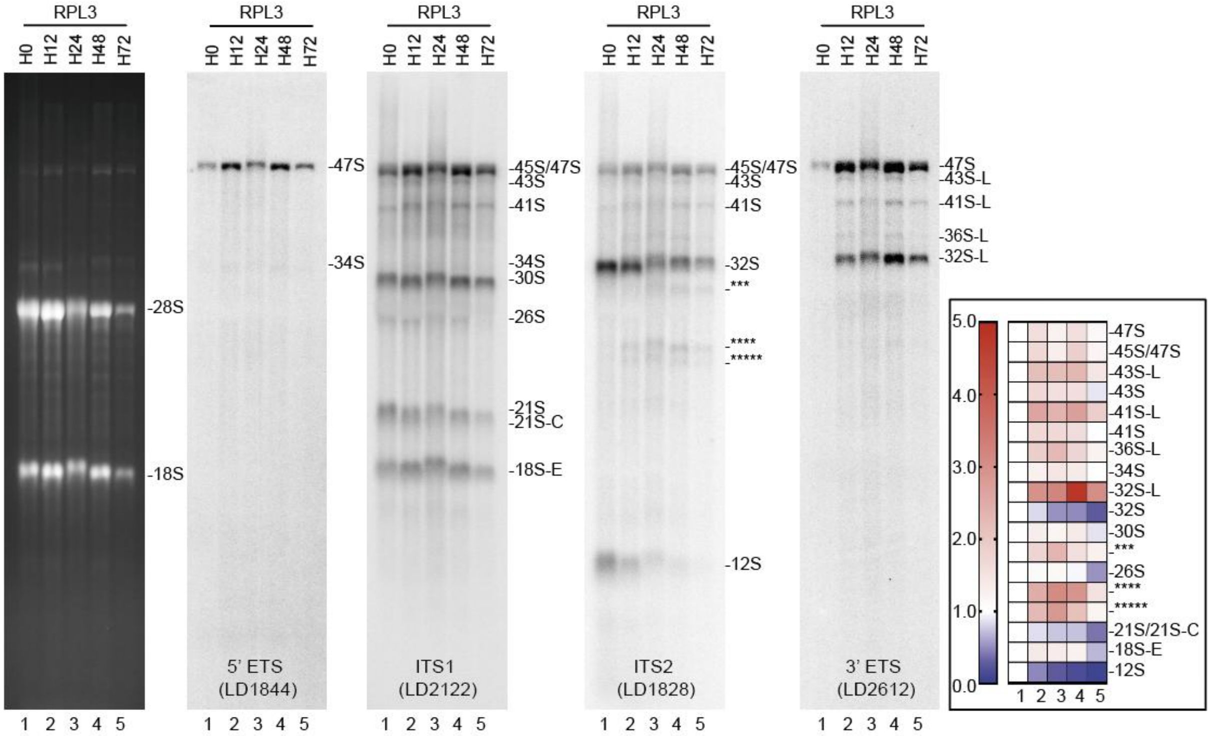

Supplementary Figure S15. Northern blots showing the optimal depletion time points for RPL3
Assessment of intermediate accumulation following processing perturbation using northern blotting. RPL3 was depleted in HEK293 cells in a time course (from 24 to up to 72 h) to identify the best time point for NanoRibolyzer analysis.

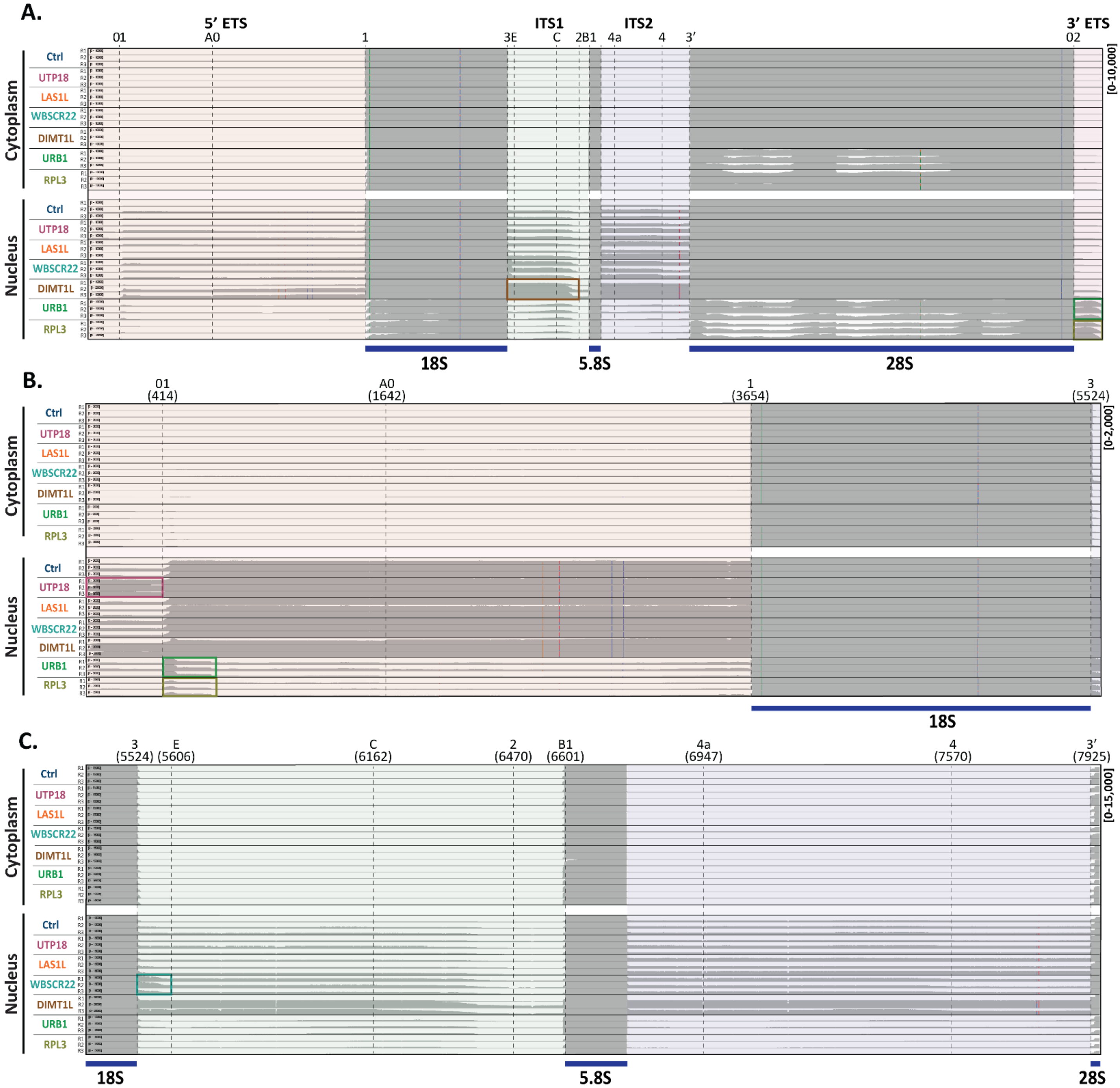

Supplementary Figure S16. IGV snapshots of nucleus/cytoplasm in knockdown samples and Ctrl

A-C, Coverage profiles of control and knockdown conditions for UTP18, WBSCR22, DIMT1L, LAS1L, URB1 and RPL3 in nucleus/cytoplasm (n=3). The profiles are shown across the entire 47S (A), 5' ETS and 18S (B), and ITS1, 5.8S, and ITS2 (C). Data ranges were normalized across all samples to visualize the coverage profiles within the selected regions. Altered regions in the selected knockdowns are highlighted in purple (UTP18 KD), turquoise (WBSCR22 KD), brown (DIMT1L KD) and green (URB1 KD), and dark yellow (RPL3 KD).

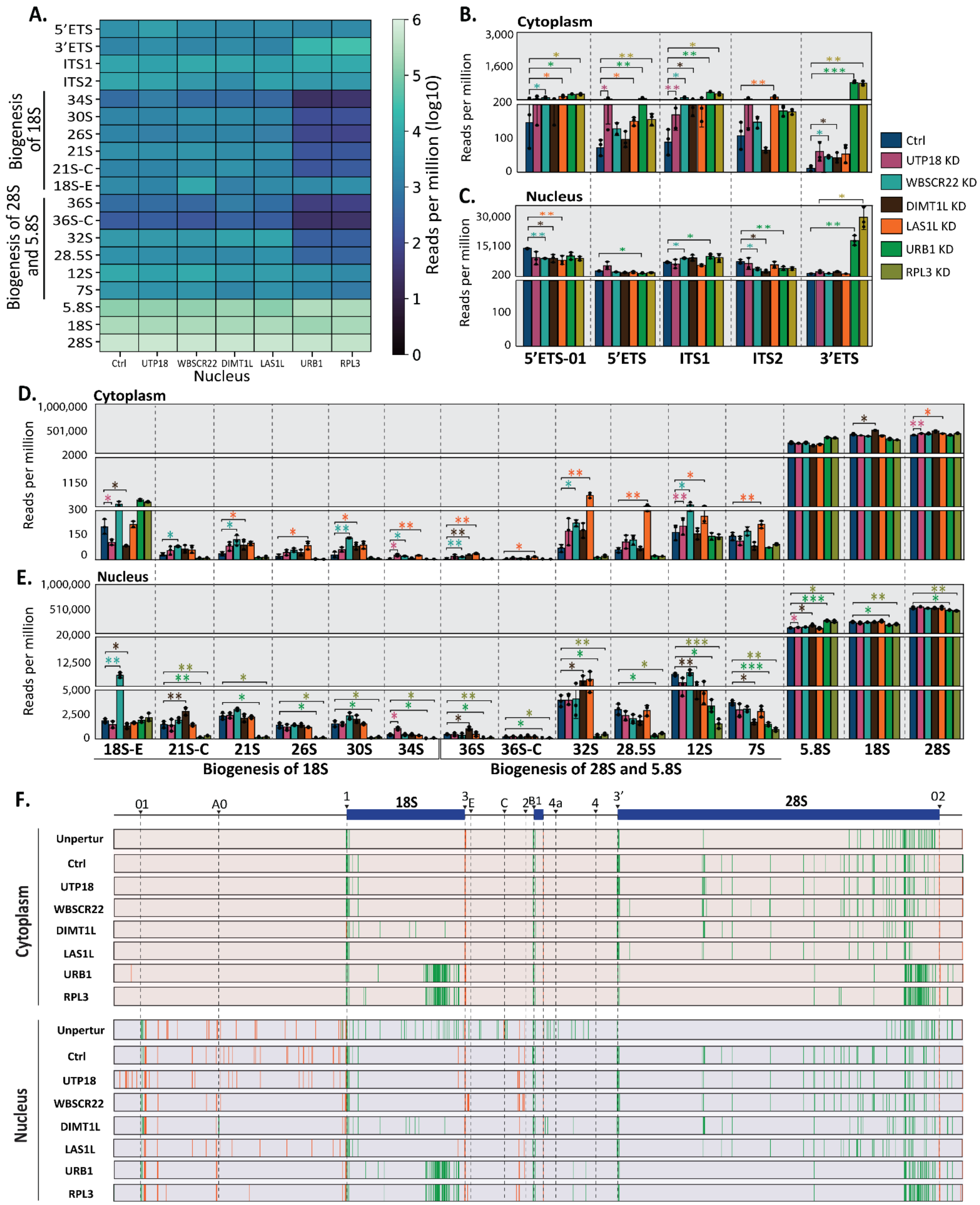

A.
5'ETS
3'ETS
ITS1
ITS2
34S
30S
26S
21S
21S-C
18S-E
36S
36S-C
32S
28.5S
12S
7S
5.8S
18S
28S
Biogenesis of 18S
Biogenesis of 28S and 5.8S
Ctrl
UTP18
WBSCR22
DIMT1L
LAS1L
URB1
RPL3
Nucleus
Reads per million (log10)
B.
Cytoplasm
Reads per million
C.
Nucleus
5'ETS-01
5'ETS
ITS1
ITS2
3'ETS
Ctrl
UTP18 KD
WBSCR22 KD
DIMT1L KD
LAS1L KD
URB1 KD
RPL3 KD
D.
Cytoplasm
E.
Nucleus
18S-E
21S-C
21S
26S
30S
34S
36S
36S-C
32S
28.5S
12S
7S
5.8S
18S
28S
Biogenesis of 18S
Biogenesis of 28S and 5.8S
F.
01
A0
1
18S
3
E
C
2
B1
4a
4
3'
28S
02
Cytoplasm
Nucleus
Unpertur
Ctrl
UTP18
WBSCR22
DIMT1L
LAS1L
URB1
RPL3

Supplementary Figure S17. Pre-rRNA intermediate quantification and visualization of cleavages sites following processing perturbations

A-B, Quantification of pre-rRNA intermediates and mature rRNAs in cytoplasm (A) and nucleus (B) of control and knockdown samples of UTP18, DIMT1L, WBSCR22, LAS1L, URB1, and RPL3 (n=3 samples each).

C-D, Quantification of external and internal transcribed spacers in cytoplasm (C) and nucleus (D) across control and knockdown samples of UTP18, DIMT1L, WBSCR22, LAS1L, URB1, and RPL3 (n=3 samples each). Scores represent reads per million, while the bar plots show the means ± SD. Statistical significance was determined using a two-tailed paired t-tests between control and KD conditions, with *$p < 0.05$, **$p < 0.01$, ***$p < 0.001$.

E, Heatmap illustrating pre-/rRNA abundance in nuclear fraction of control and UTP18, DIMT1L, WBSCR22, LAS1L, URB1 knockdowns samples, represented as log10 reads per million.

F, Visual representation of prevalent start (green) and end (orange) cleavage sites across the 47S rRNA in cytoplasmic (top) and nuclear (bottom) samples, comparing unperturbed and control samples compared to knockdown samples of UTP18, DIMT1L, WBSCR22, LAS1L, URB1, and RPL3. Each cleavage site displayed was detected in at least two out of three replicates.

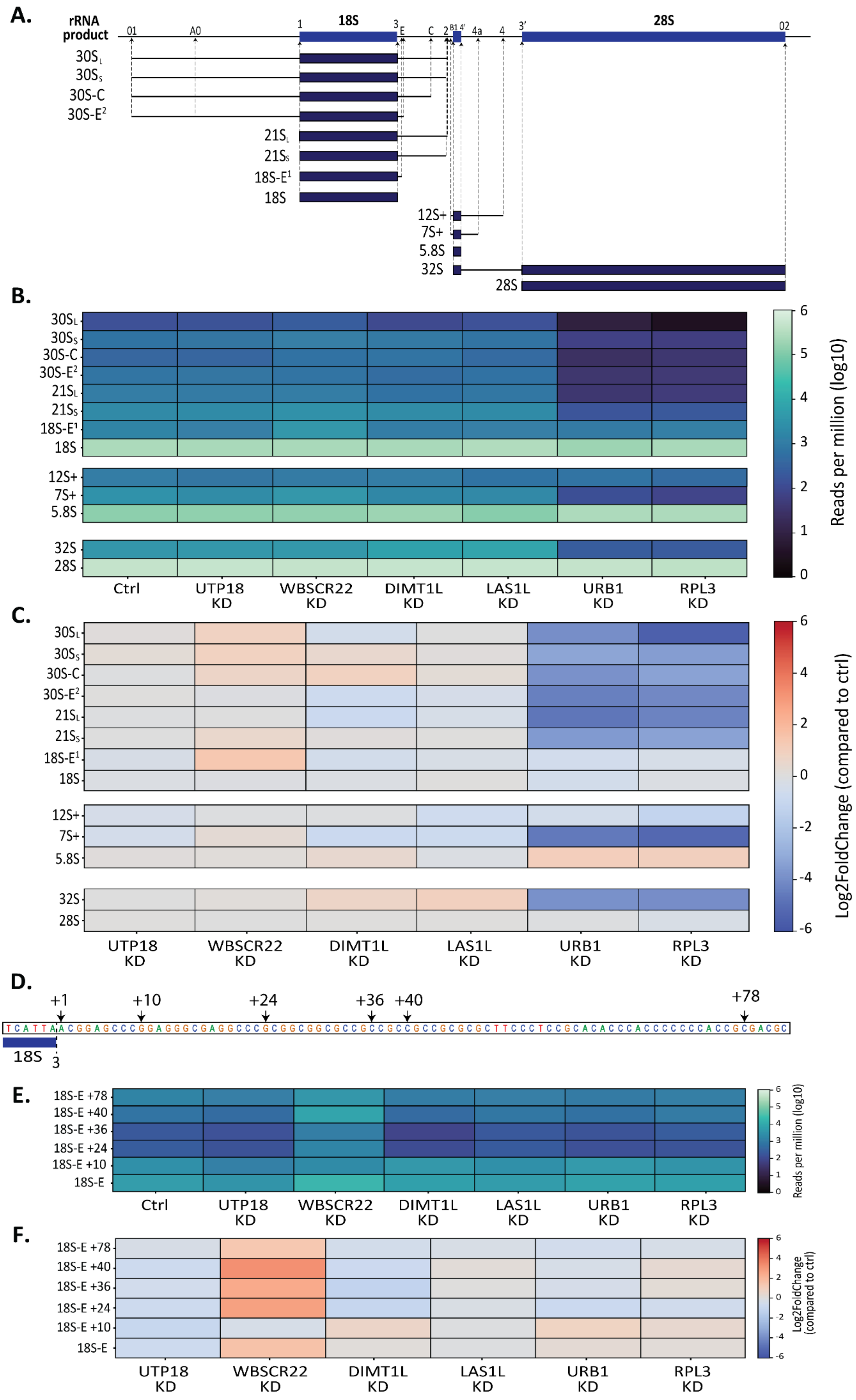
A.
rRNA product
01
A0
1
18S
3
E
C
2
4a
4
3′
28S
02
30S$_L$
30S$_S$
30S-C
30S-E[2]
21S$_L$
21S$_S$
18S-E[1]
18S
12S+
7S+
5.8S
32S
28S
B.
Ctrl
UTP18 KD
WBSCR22 KD
DIMT1L KD
LAS1L KD
URB1 KD
RPL3 KD
Reads per million (log10)
C.
Log2FoldChange (compared to ctrl)
D.
+1
+10
+24
+36
+40
+78
18S
3
E.
18S-E +78
18S-E +40
18S-E +36
18S-E +24
18S-E +10
18S-E
F.

Supplementary Figure S18. Detailed characterization of newly identified intermediates and 3' end maturation of 18S rRNA following processing perturbation

A, Illustration of the identified rRNA products in nucleus, as derived from the intensity hubs table shown in 3A.

B, Quantification of the newly detected pre-rRNA products in nuclear Ctrl and KD conditions (n=3 samples each).

C, Log2FoldChange of the newly identified pre-rRNA products in the nucleus of UTP18, DIMT1L, WBSCR22, LAS1L, URB1 and RPL3 knockdown samples compared to control (n=3 samples each).

D. Map of extended forms of 18S rRNA , including: +10, +24, +36, +40, and +78 nt extensions (termed 18S-E sub-types).

E, Quantification of the 18S-E sub-types in nuclear Ctrl and KD conditions (n=3 samples each).

F, Log2FoldChange of the 18S-E sub-types in the nucleus of UTP18, DIMT1L, WBSCR22, LAS1L, URB1 and RPL3 knockdown samples compared to control (n=3 samples each).

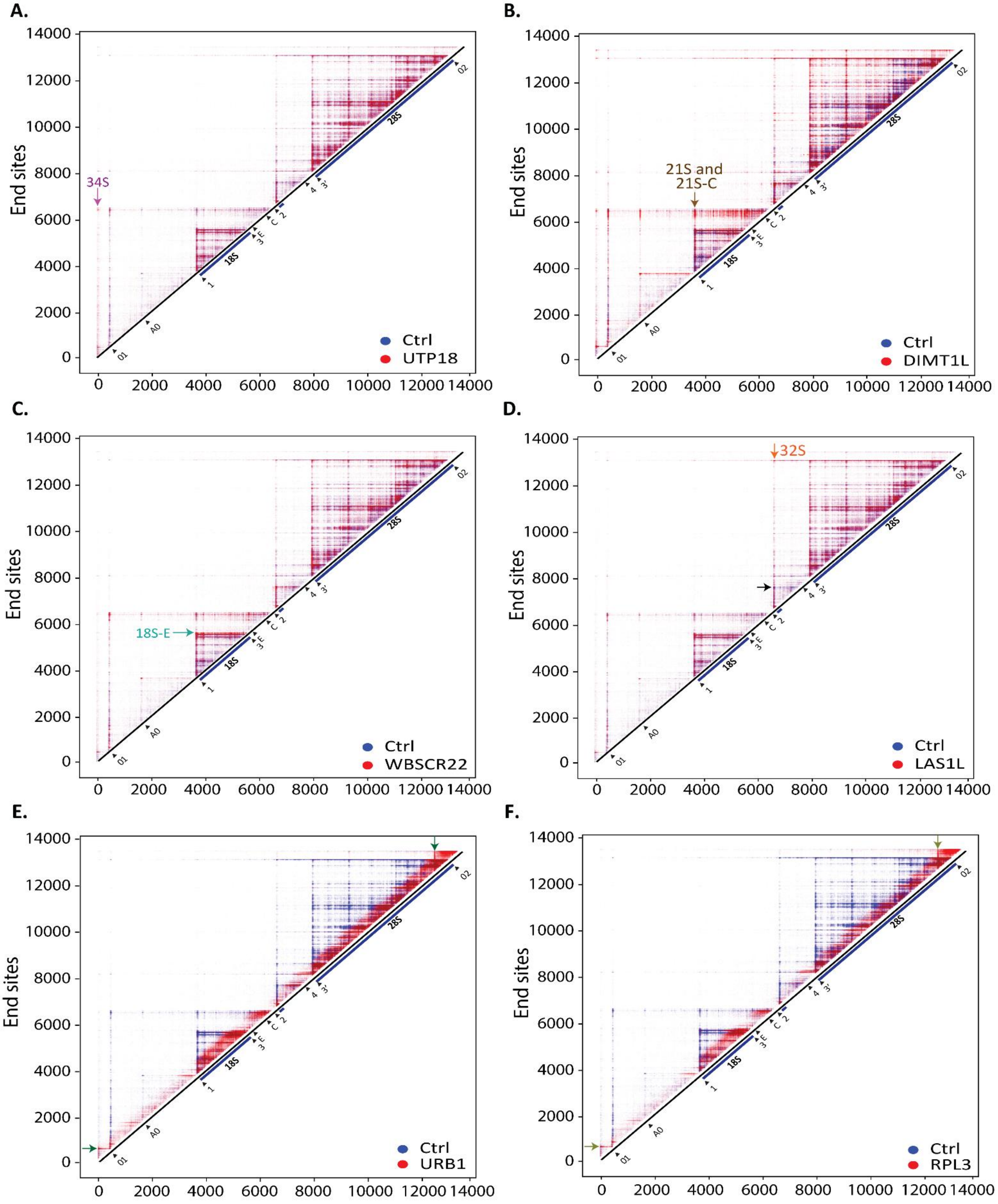

Supplementary Figure S19. Pairwise overlay of intensity matrices of processing perturbation samples

A-F,Overalyed matrices are shown in nuclear fraction nucleus between control (blue) and designated KD conditions (red) (**A** - UTP18; **B** - DIMT1L; **C** - WBSCR22; **D** - LAS1L; **E** - URB1; **F** - RPL3). Selected intensity “hubs” associated with the effected precursors are indicated with arrows colored according to KD condition shown in Figure 4B.

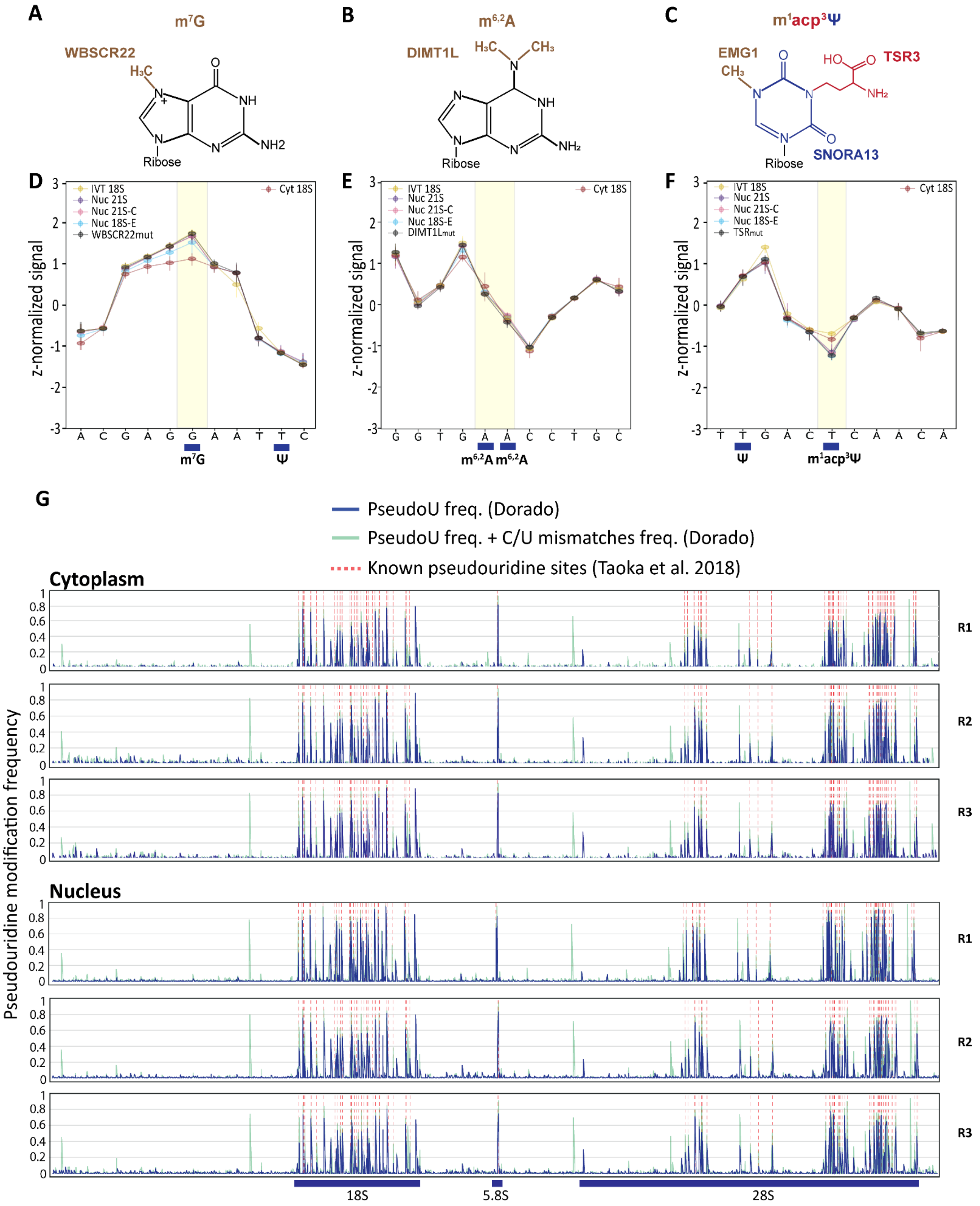

A
m7G
WBSCR22
Ribose
B
m6,2A
DIMT1L
Ribose
C
m1acp3Ψ
EMG1
TSR3
SNORA13
Ribose
D
E
F
IVT 18S
Nuc 21S
Nuc 21S-C
Nuc 18S-E
WBSCR22mut
DIMT1Lmut
TSRmut
Cyt 18S
z-normalized signal
m7G
Ψ
m6,2A m6,2A
m1acp3Ψ
G
PseudoU freq. (Dorado)
PseudoU freq. + C/U mismatches freq. (Dorado)
Known pseudouridine sites (Taoka et al. 2018)
Cytoplasm
Nucleus
Pseudouridine modification frequency
R1
R2
R3
18S
5.8S
28S

Supplementary Figure S20. Spatio-temporal precursor-specific RNA modification and Pseudouridylation modification detection along 47S

A-C, Chemical structure of RNA modifications m^7G (A), m_2^6A (B) and $m^1acp^3\Psi$ (C). WBSCR22[35] is a methyltransferase responsible for adding a methyl group at G^{1639} on 18S rRNA, DIMT1L[35] deposits m_2^6A at A^{1735} and A^{1736}, and TSR3[47] catalyzes the addition of α-amino-α-carboxyl-propyl (acp) following the pseudouridylation guided by SNORA13, m1 methylation via EMG1 at U^{1248} on 18S rRNA.

D-F, The mean and standard deviation of resquiggled, z-normalized raw current signals across 11 nucleotides flanking the modification sites on 18S rRNA are shown for m^7G (D), m_2^6A (E), and $m^1acp^3\Psi$ (F). Data is presented for nuclear precursors (21S, 21S-C, 18S-E) and mature cytoplasmic 18S rRNA. Negative controls include IVT 18S and the corresponding mutant or knockout lines (WBSCR22mut, DIMT1Lmut, TSR3mut).

G, PseudoU modification frequencies in cytoplasm (top) and nucleus (bottom) across 47S rRNA landscape (n=3 sample each). Only pseudoU frequencies (Dorardo) are shown in dark blue, summed pseudoU and C/U mismatches are shown in light green and known pseudoU sites (Taoka et al., 2018) are marked by dotted red lines. Note that sites showing only C/U mismatches without corresponding pseudouridine detection are likely onlu C/U mismatches rather than pseudouridines. For further detail in Supplementary Data 1.

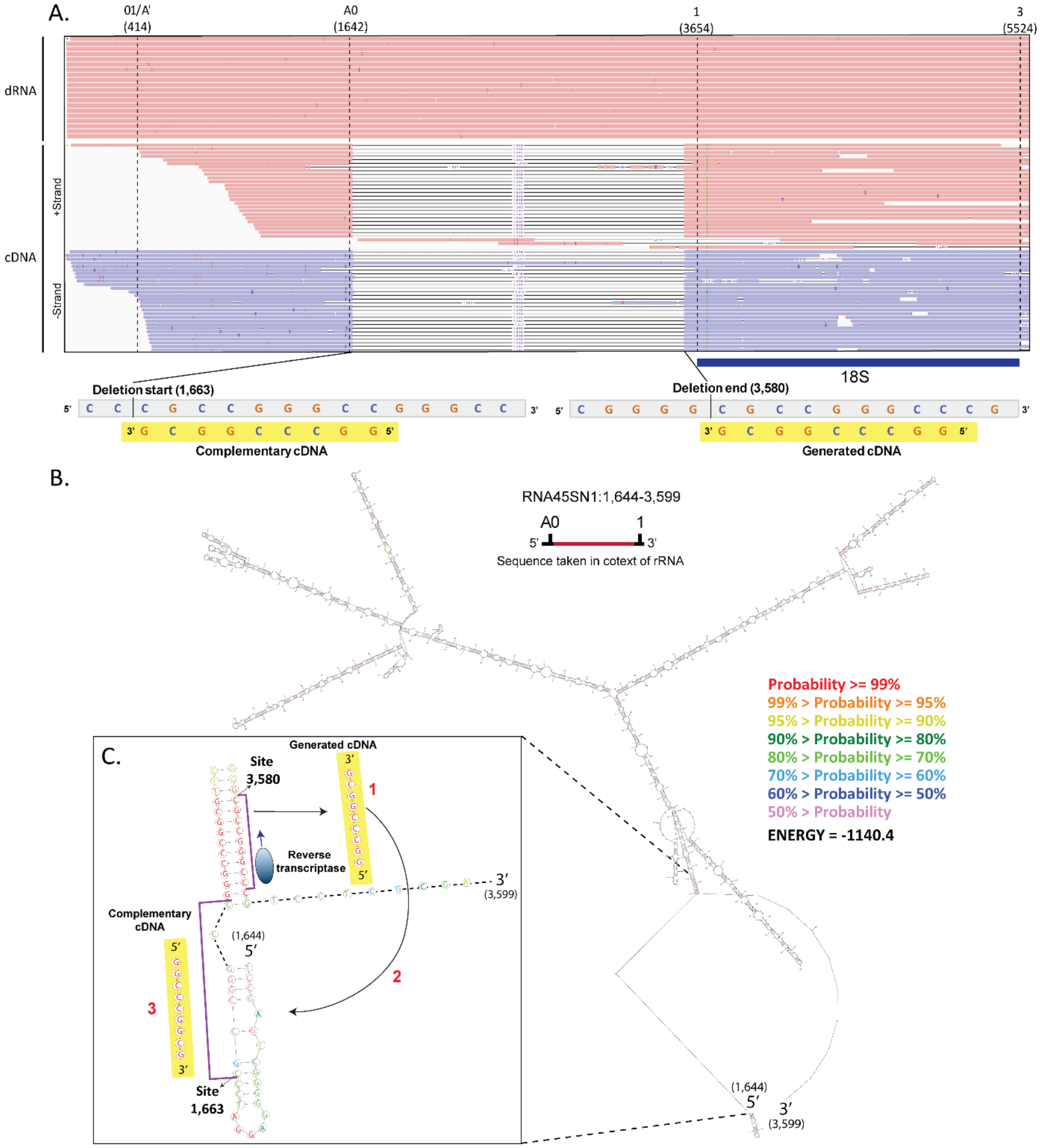

Supplementary Figure S21. Structural insights into RT-mediated stem-skipping at the 5′ ETS

A, Alignment of direct RNA (dRNA) and cDNA reads showing a systematic ~1916 bp deletion in the cDNA data between sites 1663 (5′) and 3580 (3′) of the 5'ETS. The regions flanking the deletion display sequence complementarity that could promote reannealing during reverse transcription.

B, Predicted secondary structure of the rRNA segment (positions 1644-3599) generated by RNAstructure, showing overall preserved folding with locally unpaired regions adjacent to the deletion boundaries (sites 1663 and 3580). Base-pairing probabilities are color-coded according to confidence (red ≥99%, orange 95–99%, yellow 90–95%, etc.). For more information see Reuter & Mathews (2010)[13].

C, Schematic model illustrating the proposed mechanism of reverse transcriptase RT-mediated stem-skipping. RT pauses near the 3′ end of a stem-loop (site 3580; step 1), dissociates and reanneals (step 2) to a complementary region near the 5′ side of the deletion (site 1663; step 3), then resumes synthesis, bypassing the intervening stem-loop and producing a ~1916 bp deletion.

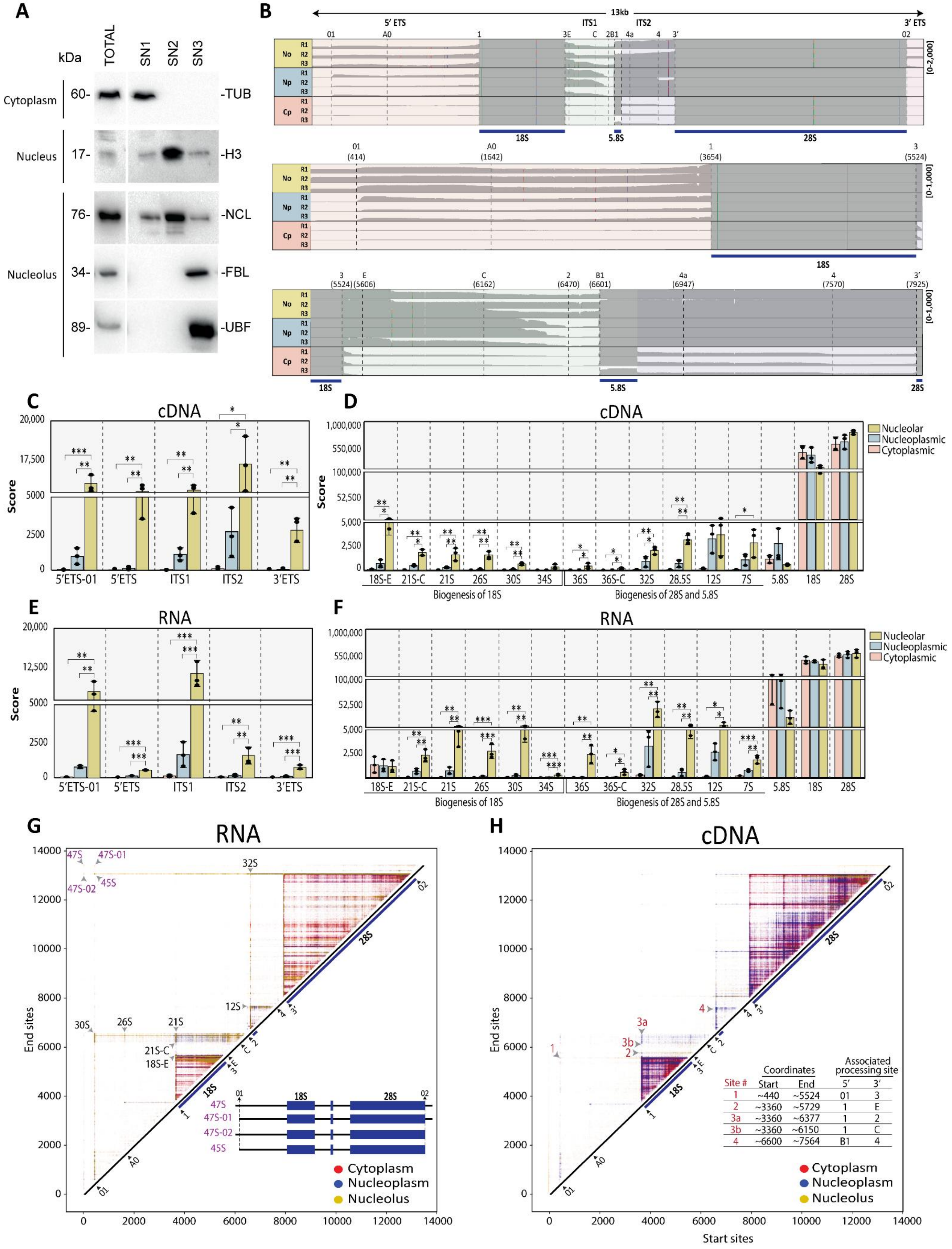
A
kDa
TOTAL
SN1
SN2
SN3
Cytoplasm
60-
-TUB
Nucleus
17-
-H3
Nucleolus
76-
-NCL
34-
-FBL
89-
-UBF
B
13kb
5′ ETS
ITS1
ITS2
3′ ETS
No
Np
Cp
R1
R2
R3
[0-2,000]
[0-1,000]
18S
5.8S
28S
01 (414)
A0 (1642)
1 (3654)
3 (5524)
E (5606)
C (6162)
2 (6470)
B1 (6601)
4a (6947)
4 (7570)
3′ (7925)
C
cDNA
Score
5′ETS-01
5′ETS
ITS1
ITS2
3′ETS
D
cDNA
Nucleolar
Nucleoplasmic
Cytoplasmic
18S-E
21S-C
21S
26S
30S
34S
36S
36S-C
32S
28.5S
12S
7S
5.8S
18S
28S
Biogenesis of 18S
Biogenesis of 28S and 5.8S
E
RNA
F
RNA
G
RNA
47S
47S-01
47S-02
45S
32S
30S
26S
21S
21S-C
18S-E
12S
End sites
Cytoplasm
Nucleoplasm
Nucleolus
H
cDNA
Start sites
Site # | Coordinates: Start | End | Associated processing site: 5′ | 3′
1 | ~440 | ~5524 | 01 | 3
2 | ~3360 | ~5729 | 1 | E
3a | ~3360 | ~6377 | 1 | 2
3b | ~3360 | ~6150 | 1 | C
4 | ~6600 | ~7564 | B1 | 4
Cytoplasm
Nucleoplasm
Nucleolus

Supplementary Figure S22. Anlaysis of pre-rRNA intermediates in cytoplasm, nucleoplasm and nucleolus

A, Western blot analysis on SN1 (Cytoplasm), SN2 (Np), and SN3 (No) fractions confirming successful fractionation. β-Tubulin (TUB) was used as a cytoplasmic marker, Histone H3 (H3) as a nuclear marker, and Nucleolin (NCL), Fibrillarin (FBL), and Upstream Binding Factor (UBF) as nucleolar markers. Marker-specific enrichment in the corresponding fractions confirms the purity and integrity of the subcellular preparations.

B, Coverage profiles of nucleolar (No), nucleoplasmic (Np) and cytoplasmic (Cp) conditions (n=3 samples each) in cDNA data. The profiles are shown across the entire 47S template (top), 5' ETS and 18S (middle), and ITS1, 5.8S, and ITS2 (bottom). Data ranges were normalized across all samples to visualize the coverage profiles within the selected regions.

C-F, Quantitative comparison of cDNA (C-D) and RNA (E-F) datasets of the ETS and ITS regions (C,E) and pre-rRNA intermediates (D,F) across the nucleolar (No), nucleoplasmic (Np) and cytoplasmic (Cp) conditions (n=3 samples each) in cDNA (top) and DRS (bottom) data. One-way ANOVA followed by Tukey test for multiple comparison post hoc test, $*p < 0.05$, $**p < 0.01$, $***p < 0.001$. Score (read per million) represents the means ± SD.

G-H, Comparison of overlayed intensity matrices of nucleolar (No), nucleoplasmic (Np) and cytoplasmic (Cp) conditions in RNA (G) and cDNA (H). Key intensity hubs and their associated precursors are marked with arrows. In G, the top left (purple) highlights the positions of 47S, 45S, and the 47S related species (47S-01 and 47S-02). In H, the tables provide detail of the site numbers of selected “hubs”, associated start and end coordinate (on 47S) and the respective 5' and 3' processing site.

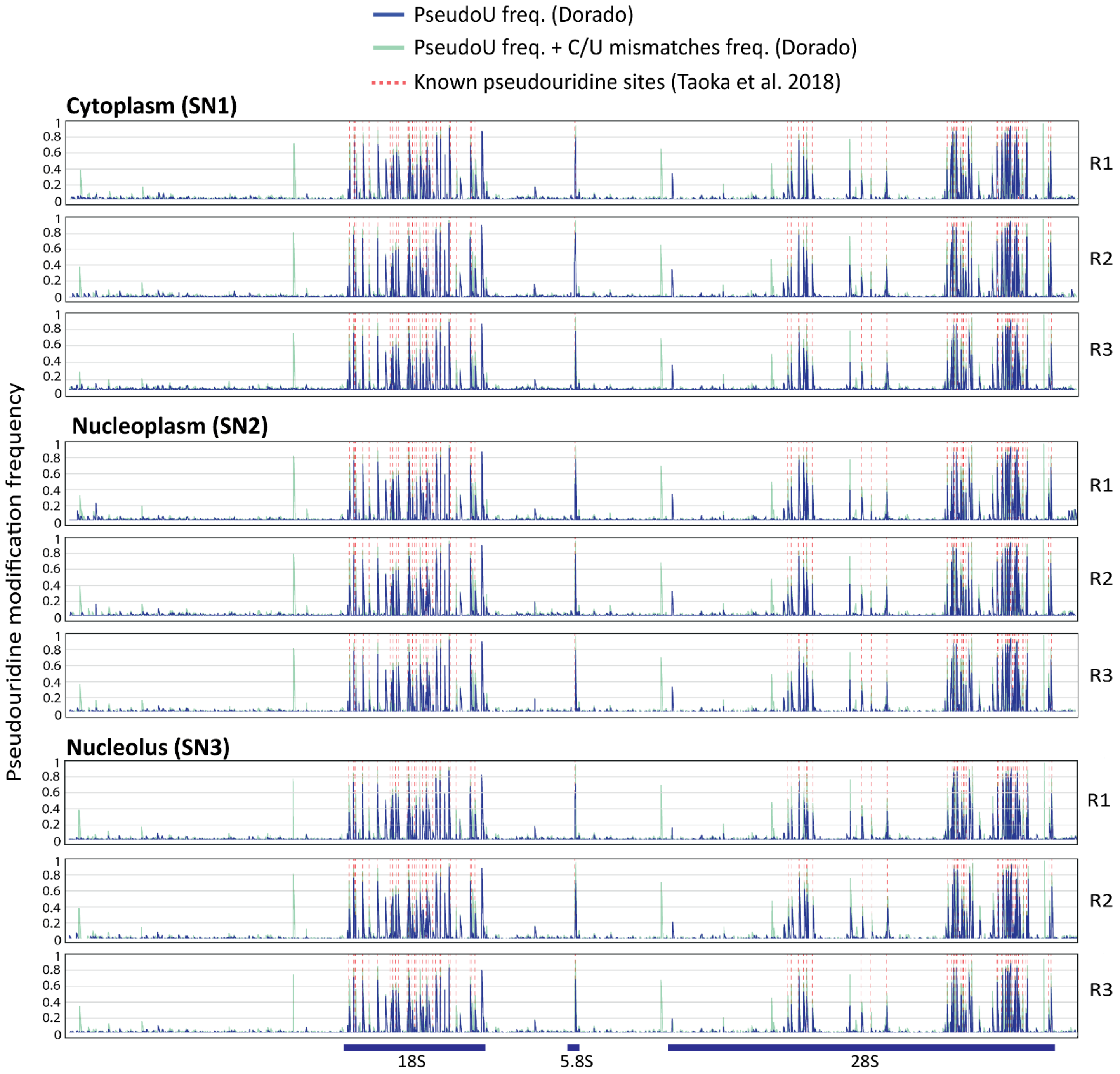

Supplementary Figure S23. Detection of pseudouridylation across full-length 47S pre-rRNA in cytoplasmic, nucleoplasmic, and nucleolar fractions using NanoRibolyzer
A, PseudoU modification frequency in cytoplasmic (top), nucleoplasmic (middle) and nucleolar (bottom) fractions across 47S rRNA (n=3 samples each). Only pseudoU frequencies (Dorado) are shown in dark blue, summed pseudoU and C/U mismatches are shown in light green and known pseudoU sites (Taoka et al., 2018) are marked by dotted red lines. Note that sites showing only C/U mismatches without corresponding pseudouridine detection are likely only C/U mismatches rather than pseudouridines. For further detail in Supplementary Data 1.

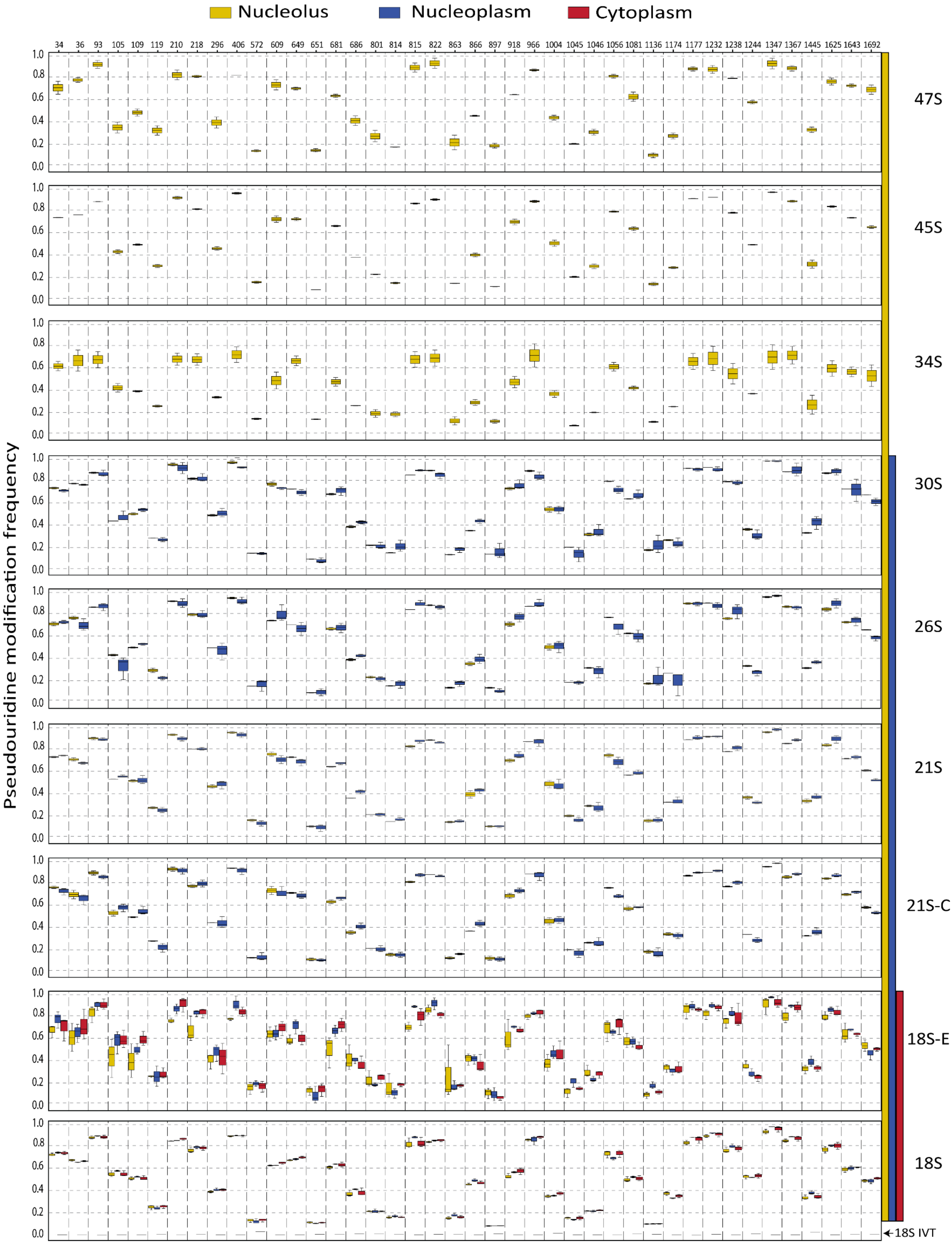

Nucleolus
Nucleoplasm
Cytoplasm
34 36 93 105 109 119 210 218 296 406 572 609 649 651 681 686 801 814 815 822 863 866 897 918 966 1004 1045 1046 1056 1081 1136 1174 1177 1232 1238 1244 1347 1367 1445 1625 1643 1692
Pseudouridine modification frequency
1.0 0.8 0.6 0.4 0.2 0.0
47S
45S
34S
30S
26S
21S
21S-C
18S-E
18S
18S IVT

Supplementary Figure S24. Pseudouridine modification frequency across 18S rRNA during ribosome biogenesis

Pseudouridine modification frequencies are shown for individual precursors during 18S rRNA maturation, spanning the nucleolar (yellow), nucleoplasmic (blue), and cytoplasmic (red) fractions. Each row represents a distinct rRNA precursor, ordered from earliest (47S) to mature 18S. The x-axis denotes the pseudouridine sites along 18S rRNA (positions indicated above). The 18S IVT serves as an unmodified negative control.

Supplementary Tables

Table S1. Normalized read count per million (CPM) of Xist and Malat1 transcripts in cytoplasmic, whole cell, and nuclear fractions (n=3 samples each). Fold change (FC) between the conditions is shown above each comparison.

Normalized counts

Gene id	ENSG00000229807.13	ENSG00000251562.10
**gene_name**	**XIST**	**MALAT1**
**Cytoplasm1_cpm**	395.34	20.81
**Cytoplasm2_cpm**	302.24	65.70
**Cytoplasm3_cpm**	408.33	72.06
**Cell1_cpm**	4080.08	464.70
**Cell2_cpm**	2407.99	210.92
**Cell3_cpm**	2060.61	281.88
**Nucleus1_cpm**	69789.40	7540.67
**Nucleus2_cpm**	64370.22	6857.48
**Nucleus3_cpm**	49221.52	7795.12

Foldchange(FC)

gene	XIST	MALAT1
**nuc_vs_cell**	21.45141	23.17845
**nuc_vs_cyt**	165.8204	139.9601
**cell_vs_cyt**	7.730049	6.038373

Table S2. Silencer RNA sequence siRNA inactivation experiments.

Target (Lab ID)	Silencer RNA sequence
UTP18(#30)	CAUUCUCUUCAGAUAGUAAtt
	UUACUAUCUGAAGAGAAUGtg
WBSR22 (#20)	GAGUGGAAGUUAUCUGUCAtt
	UGACAGAUAACUUCCACUCag
DIMT1L (#69)	GGCUAGUAGCUGAACUUCAtt
	UGAAGUUCAGCUACUAGCCtt
LAS1L #139)	UGAAGUUCAGCUACUAGCCtt
	AAUGGAACAGAUGCGCAGCag
URB1 (#859)	rCrUrArCrUrUrUrArCrCrUrGrUrUrGrGrArUrGrUrArGrUCC
	rGrGrArCrUrArCrArUrCrCrArArCrArGrGrUrArArArGrUrArGrCrU
RPL3 (#857)	rGrUrGrArCrCrArArGrGrGrCrArArArGrGrCrUrArCrArAAG
	rCrUrUrUrGrUrArGrCrCrUrUrUrGrCrCrCrUrUrGrGrUrCrArCrCrC
RPS26 (#937)	rCrArU rUrUrC rUrGrA rArGrC rGrArG rCrGrU rCrUrU rCrGAT
RPS26	rArUrC rGrArA rGrArC rGrCrU rCrGrC rUrUrC rArGrA rArArUrGrUrC

Table S3. Probes for northern blotting in this study.

**Lab IDs**	**Targeted area**	**sequence**
LD1844	5’ ETS	CGGAGGCCCAACCTCTCCGACGACAGGTCGCCAGAGGACAGCGTGTCAGC
LD2122	ITS1	GCCCTCCGGGCTCCGTTAATGATC
LD1828	ITS2	CTGCGAGGGAACCCCCAGCCGCGCA
LD2612	3’ ETS	GAGGAGGCGGGAACCGAAGAAGCGG

Supplementary Notes

Supplementary Note S1. PseudoU basecaller

Our data reliably reproduced and identified all reported pseudouridines (pseudoU) sites from the reference study by Taoka et al. (2018)[14]. However, accurate quantification of pseudoU levels remains a challenge. Multiple studies have noted that pseudoU modifications can frequently produce U-to-C mismatches during sequencing[15,16,17,18,19]. This issue complicates pseudoU quantification, as it relies on a two-step analysis: first, basecalling from pod5 files identifies nucleotide composition (i.e. U), and then Remora assesses modification status to confirm pseudoU sites within basecalled U. Yet, if some pseudoU are misbasecalled as C, they bypass the Remora model, resulting in underrepresentation of pseudoU. A potential approach to more accurately estimate pseudoU levels is to utilize the U-to-C mismatch ratio as an indicator of potential pseudoU. By summing up the U-to-C mismatch rate with the identified pseudoU, we can derive a more realistic, though not precise, estimate of pseudoU presence at a given site[18]. Additionally, recent work has demonstrated that accurate quantification of pseudouridylation by ONT direct-RNA sequencing strongly depends on the sequence context around the modification and that signal deviations from Ψ extend beyond the canonical 5-mer window[17]. With continued improvements in basecalling models, these limitations are likely to be resolved, enabling more reliable and easily generated datasets for studying RNA modifications.

Supplementary Note S2. Differences between DRS and cDNA-sequencing

In this work, we employ both cDNA sequencing and direct RNA sequencing (DRS) to capture pre-rRNAs and mature rRNAs. cDNA sequencing enables cost-effective multiplexing of samples, allowing for the inclusion of replicates for statistical analysis. In contrast, DRS was primarily utilized for detection RNA modifications at the signal level and using pseudoU basecaller. Additionally, we employed a recently developed barcoding strategy for DRS (SeqTagger), allowing us to multiplex up to four samples per flow cell, a capability that was not available until recently[20]. Thus, this approach also provides a cost-effective solution for DRS, with multiple replicates within the same flow cell. While cDNA and DRS approaches yielded largely reproducible results (Fig 5B), slight variations were observed, likely due to differences in library preparation of each method. For instance, for cDNA libraries, Maxima H Minus RT (Thermo Fisher) was used at 42°C (ONT protocol) to synthesize first-strand cDNA with a 5′ CCC overhang, enabling barcoding and high-throughput sequencing. In contrast, DRS employed the recently developed Induro RT (NEB), a thermostable enzyme capable of reverse transcription at 55°C, improving the yield, read length, and resolution of structured RNAs like pre-rRNA[21]. This enhanced performance likely enabled better capture of long precursors such as 47S (~13 kb), 45S, and novel variants 47S-01 and 47S-02, particularly evident in unsupervised approach. Although Induro RT can generate very long cDNA fragments, it has not yet been coupled with second-strand synthesis for barcoded, high-throughput cDNA sequencing. Future work should explore this potential to further expand sequencing capabilities.

References

1. Tomecki, R., Sikorski, P. J., & Zakrzewska-Placzek, M. (2017). Comparison of preribosomal RNA processing pathways in yeast, plant and human cells – focus on coordinated action of endo- and exoribonucleases. FEBS Letters, 591(13), 1801–1850.

2. Henras, A. K., Plisson-Chastang, C., O'Donohue, M. F., Chakraborty, A., & Gleizes, P. E. (2015). An overview of pre-ribosomal RNA processing in eukaryotes. Wiley Interdisciplinary Reviews: RNA, 6(2), 225–242.

3. Mullineux, S. T., & Lafontaine, D. L. J. (2012). Mapping the cleavage sites on mammalian pre-rRNAs: where do we stand? Biochimie, 94(7), 1521–1532.

4. Kass, S., Craig, N., & Sollner Webbi, B. (1987). Primary Processing of Mammalian rRNA Involves Two Adjacent Cleavages and Is Not Species Specific. MOLECULAR AND CELLULAR BIOLOGY, 7(8), 2891–2898.

5. Rouquette, J., Choesmel, V., & Gleizes, P. E. (2005). Nuclear export and cytoplasmic processing of precursors to the 40S ribosomal subunits in mammalian cells. The EMBO Journal, 24(16), 2862–2872.

6. Preti, M., O'Donohue, M. F., Montel-Lehry, N., Bortolin-Cavaillé, M. L., Choesmel, V., & Gleizes, P. E. (2013). Gradual processing of the ITS1 from the nucleolus to the cytoplasm during synthesis of the human 18S rRNA. Nucleic Acids Research, 41(8), 4709.

7. Montellese, C., Montel-Lehry, N., Henras, A. K., Kutay, U., Gleizes, P. E., & O'Donohue, M. F. (2017). Poly(A)-specific ribonuclease is a nuclear ribosome biogenesis factor involved in human 18S rRNA maturation. Nucleic Acids Research, 45(11), 6822–6836.

8. Goldfarb, K. C., & Cech, T. R. (2017). Targeted CRISPR disruption reveals a role for RNase MRP RNA in human preribosomal RNA processing. Genes and Development, 31(1), 59–71.

9. Idol, R. A., Robledo, S., Du, H.-Y., Crimmins, D. L., Wilson, D. B., Ladenson, J. H., Bessler, M., & Mason, P. J. (2007). Cells depleted for RPS19, a protein associated with Diamond Blackfan Anemia, show defects in 18S ribosomal RNA synthesis and small ribosomal subunit production.

10. Ishikawa, H., Yoshikawa, H., Izumikawa, K., Miura, Y., Taoka, M., Nobe, Y., Yamauchi, Y., Nakayama, H., Simpson, R. J., Isobe, T., & Takahashi, N. (2017). Poly(A)-specific ribonuclease regulates the processing of small-subunit rRNAs in human cells. Nucleic Acids Research, 45(6), 3437–3447.

11. Schillewaert, S., Wacheul, L., Lhomme, F., & Lafontaine, D. L. J. (2012). The Evolutionarily Conserved Protein LAS1 Is Required for Pre-rRNA Processing at Both Ends of ITS2. Molecular and Cellular Biology, 32(2), 430.

12. Sloan, K. E., Mattijssen, S., Lebaron, S., Tollervey, D., Pruijn, G. J. M., & Watkins, N. J. (2013). Both endonucleolytic and exonucleolytic cleavage mediate ITS1 removal during human ribosomal RNA processing. Journal of Cell Biology, 200(5), 577–588.

13. Reuter, J. S., & Mathews, D. H. (2010). RNAstructure: Software for RNA secondary structure prediction and analysis. BMC Bioinformatics, 11(1), 129.

14. Taoka, M., Nobe, Y., Yamaki, Y., Sato, K., Ishikawa, H., Izumikawa, K., Yamauchi, Y., Hirota, K., Nakayama, H., Takahashi, N., & Isobe, T. (2018). Landscape of the complete RNA chemical modifications in the human 80S ribosome. Nucleic Acids Research, 46(18), 9289–9298.

15. Begik, O., Diensthuber, G., Liu, H., Delgado-Tejedor, A., Kontur, C., Niazi, A. M., Valen, E., Giraldez, A. J., Beaudoin, J. D., Mattick, J. S., & Novoa, E. M. (2022). Nano3P-seq: transcriptome-wide analysis of gene expression and tail dynamics using end-capture nanopore cDNA sequencing. *Nature Methods 2022 20:1*, *20*(1), 75–85.

16. Tavakoli, S., Nabizadeh, M., Makhamreh, A., Gamper, H., McCormick, C. A., Rezapour, N. K., Hou, Y. M., Wanunu, M., & Rouhanifard, S. H. (2023). Semi-quantitative detection of pseudouridine modifications and type I/II hypermodifications in human mRNAs using direct long-read sequencing. *Nature Communications*, *14*(1), 334.

17. Makhamreh, A., Tavakoli, S., Fallahi, A., Kang, X., Gamper, H., Nabizadehmashhadtoroghi, M., Jain, M., Hou, Y. M., Rouhanifard, S. H., & Wanunu, M. (2024). Nanopore signal deviations from pseudouridine modifications in RNA are sequence-specific: quantification requires dedicated synthetic controls. *Scientific Reports 2024 14:1*, *14*(1), 1–13.

18. Hewel, C., Hofmann, F., Dietrich, V., Wierczeiko, A., Friedrich, J., Jenson, K., Mündnich, S., Diederich, S., Sys, S., Schartel, L., Schweiger, S., Helm, M., Lemke, E. A., Linke, M., & Gerber, S. (2024). Direct RNA sequencing (RNA004) allows for improved transcriptome assessment and near real-time tracking of methylation for medical applications. *BioRxiv*, 2024.07.25.605188.

19. Schartel, L., Jann, C., Wierczeiko, A., Butto, T., Mündnich, S., Marchand, V., Motorin, Y., Helm, M., Gerber, S., & Lemke, E. A. (2024). Selective RNA pseudouridinylation in situ by circular gRNAs in designer organelles. *Nature Communications 2024 15:1*, *15*(1), 1–10. https://doi.org/10.1038/s41467-024-53403-1

20. Pryszcz, L. P., Diensthuber, G., Llovera, L., Medina, R., Delgado-Tejedor, A., Cozzuto, L., Ponomarenko, J., & Novoa, E. M. (2025). Rapid and accurate demultiplexing of direct RNA nanopore sequencing data with SeqTagger. Genome Research, 35(4), 956–966. https://doi.org/10.1101/GR.279290.124/-/DC1

21. Zeglinski, K., Montellese, C., Ritchie, M. E., Alhamdoosh, M., Vonarburg, C., Bowden, R., Jordi, M., Gouil, Q., Aeschimann, F., & Hsu, A. (2024). An optimized protocol for quality control of gene therapy vectors using nanopore direct RNA sequencing. Genome Research, 34(11), 1966–1975. https://doi.org/10.1101/GR.279405.124
