# Supplementary material for: Mapping human pre-rRNA processing and modification at single nucleotide resolution using long read nanopore sequencing
Source: Nat Commun. 2026 Mar 31;17:4658. doi: 10.1038/s41467-026-71164-x (PMC13201660; doi:10.1038/s41467-026-71164-x)
Supplement: Supplementary file 2 — Description of Additional Supplementary Files [file 41467_2026_71164_MOESM2_ESM.docx]

Description of Additional Supplementary Files

File name: Supplementary Data 1

Description: Pseudouridine sites detected using ONT pseudoU basecaller across 19 DRS datasets
